# Significance and Relevance of Spermatozoal RNAs to Male Fertility in Livestock

**DOI:** 10.3389/fgene.2021.768196

**Published:** 2021-12-09

**Authors:** Bijayalaxmi Sahoo, Ratan K. Choudhary, Paramajeet Sharma, Shanti Choudhary, Mukesh Kumar Gupta

**Affiliations:** ^1^ Department of Biotechnology and Medical Engineering, National Institute of Technology Rourkela, Rourkela, India; ^2^ College of Animal Biotechnology, Guru Angad Dev Veterinary and Animal Sciences University, Ludhiana, India

**Keywords:** circRNA, lncRNA, ncRNA, piRNA, spermatozoa, spermatozoal RNA, spRNA, transcriptome

## Abstract

Livestock production contributes to a significant part of the economy in developing countries. Although artificial insemination techniques brought substantial improvements in reproductive efficiency, male infertility remains a leading challenge in livestock. Current strategies for the diagnosis of male infertility largely depend on the evaluation of semen parameters and fail to diagnose idiopathic infertility in most cases. Recent evidences show that spermatozoa contains a suit of RNA population whose profile differs between fertile and infertile males. Studies have also demonstrated the crucial roles of spermatozoal RNA (spRNA) in spermatogenesis, fertilization, and early embryonic development. Thus, the spRNA profile may serve as unique molecular signatures of fertile sperm and may play pivotal roles in the diagnosis and treatment of male fertility. This manuscript provides an update on various spRNA populations, including protein-coding and non-coding RNAs, in livestock species and their potential role in semen quality, particularly sperm motility, freezability, and fertility. The contribution of seminal plasma to the spRNA population is also discussed. Furthermore, we discussed the significance of rare non-coding RNAs (ncRNAs) such as long ncRNAs (lncRNAs) and circular RNAs (circRNAs) in spermatogenic events.

## Introduction

Spermatogenic defects and sperm abnormalities are responsible for high incidence of male infertility cases in both animals and human. The diagnosis and treatment of spermatogenic failure remain to be a thrilling challenge to veterinarians and medical practitioners despite significant research progress in the investigation and treatment of infertility. Male infertility in livestock is commonly evaluated from semen quality assessment parameters such as sperm concentration, forward progressive motility, morphological defects, acrosomal abnormalities, hypo-osmotic swelling test (HOST) for membrane integrity, etc. Numerous functional assays have also been developed to evaluate the competence of spermatozoa within the female genital tract and include assessment of *in vitro* capacitation, acrosomal reaction and hypermotility, cervical mucus penetration test, zona-free hamster egg penetration assay, and *in vitro* fertilization (IVF) (Saacke et al., 2000; Foxcroft et al., 2008). Assessment of DNA fragmentation in the sperm nucleus has been a relatively recent addition to the semen evaluation parameters in farm animals (Kumaresan et al., 2020). However, these procedures fail to diagnose spermatogenic failure and, several cases remain undiagnosed and declared as idiopathic. Heterogeneous sperm population within a single ejaculate, seasonal variation, influence of semen freezing protocol, etc., further adds to the variability of results (Yang et al., 2010; Varona et al., 2019). Thus, there is a need to develop newer molecular and biochemical methods for rapid and reliable diagnosis of spermatogenic failure and male infertility.

Spermatozoa contain a suite of RNA species, including both coding RNAs and non-coding RNAs such as microRNAs (miRNAs), intranuclearRNAs, small nucleolar RNAs (snRNAs), etc. The concentration of spermatozoal RNA (spRNA) may be as much as 0.015 pg per sperm (in humans), which is conspicuously high considering the high nuclear: cytoplasmic ratio and very low volume of cytoplasm in sperm (Miller et al., 2005). Microarray analysis has revealed that more than 3,500 unique mRNAs were present in ejaculated human spermatozoa (Ostermeier et al., 2002). However, these spRNAs have traditionally been considered a residual of the spermatogenesis process due to transcriptionally dormant nuclear genome (Hecht, 1998) and lack of 28S rRNA and 18S rRNA in the cytoplasm of mammalian spermatozoa (Miller et al., 1999). The lack of rRNAs eliminates the possibility of translation within sperm and hence, *de novo* protein synthesis (Miller et al., 1999).

More recent studies have shown that, during fertilization, at least six sperm-specific, developmentally related mRNAs are delivered to the zygote (Ostermeier et al., 2004). Studies have further shown that protein-coding spRNAs undergo spatio-temporally regulated degradation during early embryonic development (Ziyyat, 2001; Hayashi, 2003; Ostermeier et al., 2004). Thus, spRNAs may have specific functions during early embryonic development, although their exact role or mechanism of action remains elusive. (Wykes et al., 1997) observed that spRNA were localized at the periphery of the sperm nucleus and co-localized with spermatozoal histone. Since imprinted genes such as *IGF2* are bound with histone protein in the sperm nucleus, it has been suggested that spRNAs may have a role in meditating imprinting mechanism or chromatin repackaging following fertilization (Miller et al., 2005). Microarray profiling of spRNAs in infertile men revealed a distinct RNA profile that could be used as a molecular signature of infertile man’s sperm (Ostermeier et al., 2002) (Wang et al., 2004). Thus, knowing the identity, functions, and regulation of spRNAs may have direct relevance to the diagnosis of male infertility and developing RNA-based therapies or contraceptives. The aberrance or absence of spRNA may be a contributing factor for idiopathic infertility and may bear on the poor performance of livestock. However, while many studies have analyzed the spRNA population in human spermatozoa, there are extremely limited results from livestock species. This review article provides a detailed review of known spRNA populations in various livestock species, their possible role in male fertility and early embryonic development, and their significance in the diagnosis and/or treatment of idiopathic infertility. A brief on RNA populations in seminal plasma is also discussed. Owning to very limited data on spRNAs in livestock, suitable relevance is also taken from humans.

### Spermatogenesis and Spermatozoa

Spermatogenesis is a complex set of events initiated at the germinal epithelium of seminiferous tubules. The spermatogonial stem cells (SSCs), the stem cells at the basement of germinal epithelium, are responsible for the maintenance of spermatogenesis throughout the adulthood of males. They possess the ability to self-renew themselves and differentiate into haploid spermatozoa through the well-orchestrated spermatogenesis process. Upon molecular cues to differentiate, the SSCs initially divide mitotically to produce spermatogonia and subsequently undergo a meiotic process to produce spermatocytes, followed by the production of haploid spermatids (Gomes et al., 2013). The spermatids undergo sequential morphological changes by spermiogenesis to develop into haploid spermatozoa. The spermatozoa in seminiferous tubules are immature and undergo a maturation process in the epididymis. The epididymal maturation of spermatozoa is characterized by several physiological and molecular changes in the plasma membrane (e.g., lipid composition, surface proteins, etc.) and nucleus (e.g., DNA condensation, protamine formation, chromatin rearrangement, etc.) (Légaré et al., 2017), formation of acrosome and development of flagella, etc. before spermiation (Griswold, 2016; Neto et al., 2016). Due to cytoplasmic expulsion, mature spermatozoa are produced with very little-to-no cytoplasm. During sperm maturation, sperm transition proteins (TNP1 and TNP2) replaces the majority of the histone proteins of the spermatids, followed by the deposition of highly basic protamines (PRM1 and PRM2) in elongated spermatids and spermatozoa (Jambor et al., 2017). The complex chromatin packaging makes the sperm epigenome highly stable and makes it transcriptionally inactive (Lambard et al., 2004). However, transcriptional and translational activities have been observed during the early stages of spermiogenesis (Miller, 2007; Yang et al., 2010) until the development of round spermatids. Translation of specific mRNAs also continues for several days after discontinuing the transcription process and completing spermiogenesis. The latter is believed to occur from those spRNAs that are associated with leftover histone protein in the nucleus and are potentiated for transcription (Miller, 2007).

### The spRNAs Population in Spermatozoa

**FIGURE 1:**
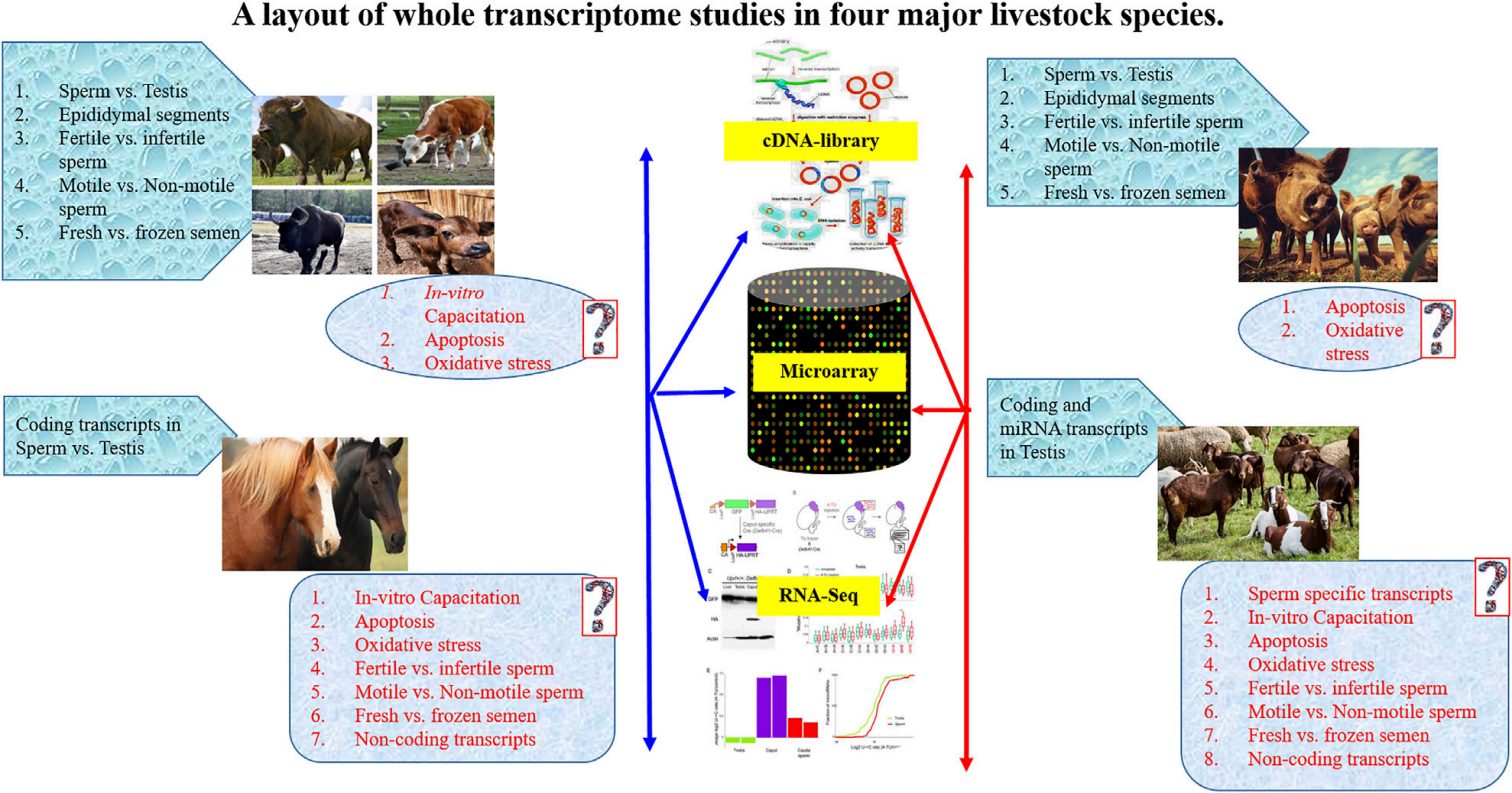
A layout of whole transcriptome studies in four major livestock species.

In last 1 decade, a number of studies have prepared cDNA libraries and performed high-thoroughput RNA sequencing (RNA-seq) or microarray analysis of spermatozoal samples from epididymis and ejaculates. The spermatozoal samples have been compared between fertilie and infertile males for spRNA profiling (Figure 1) and reported to have several coding and non-coding mRNA transcripts (Li and Zhou, 2012). The concentration of spRNA varied from picograms to femtograms per cell ranging from 100 fg in mice (Miller, 2014), 10–20 fg in humans (Zhang et al., 2017), two fg in bull (Sellem et al., 2020), 5 fg in swine (Hamatani, 2012), to 2–20 fg in stallions (Das et al., 2010). However, earlier reports on spRNAs have suggested their inertness for transcriptional and translational activities in spermatozoa (Zhang et al., 2017). The mature spermatozoa contain insufficient 28S or 18S rRNAs to support translation (Miller et al., 1999). The absence of essential components of translational machinery raised scepticism on *de novo* translation or any possible alternative roles of spRNAs in spermatozoa beyond the delivery of a paternal genome. Thus, the presence of spRNA not only aroused controversies but is intriguing to date from the discovery of sperm-borne RNAs in zygotes (Krawetz et al., 2011). The presence of RNAs in the male gamete is traditionally assumed to be either degraded leftover or spermatogenic expulsion of the residual body (Zhang et al., 2017). It was believed that *de novo* gene expression could not occur in mature spermatozoa due to highly compacted DNA by protamine that substitutes histone proteins during spermiogenesis (Balhorn, 2007). Conversely, spermatozoa rely upon pre-formed spRNAs and proteins for chromatin re-compaction, completion of the spermatogenesis process, and subsequent fertilization events (Miller, 2014).

Although sperm RNA population consists of various classes of coding RNAs, non-coding RNAs (miRNAs, piRNAs, siRNAs, lncRNAs), mitochondrial RNAs, ribosomal RNAs (rRNAs), and some intronic retained elements (Miller, 2007), the accuracy in determining its quantity and functional significance has been challenging (Dadoune, 2009). It was suggested that the variable amount of RNA in spermatozoa in different species might be due to differences in RNA isolation protocol or contamination of somatic cells in the semen. Thus, an optimized protocol for sperm purification and RNA extraction is required before any conclusion being drawn on their relevancy to male fertility (Gòdia et al., 2018). Nevertheless, evidence of both coding and non-coding spRNAs were proclaimed in different livestock species, including cattle, pigs, and stallion (Kempisty et al., 2008). Notably, full-length mRNA transcripts have also been reported (Sun et al., 2021) that had the potential to be translated *de novo* under certain circumstances (Miller and Ostermeier, 2006).

The spRNA was also suggested to have the potential to modulate phenotype through epigenetic alternations in gene expression (Pantano et al., 2015) and imprinting of IGF2 expression (Boerke et al., 2007). It was shown that spRNAs were located at the periphery of the nucleus at the boundary of histone-bound and protamine-bound DNA in the sperm. Thus, they may have an association with potentiated DNAs for gene expression (Jodar et al., 2013). Further, the sperm transcriptome profiling using Microarray (Das et al., 2013; Zhang Y. et al., 2017), and RNA-seq (Gòdia et al., 2018) have identified a suite of RNAs in spermatozoa (Sendler et al., 2013) that included both coding and non-coding transcripts and were associated with regulation of various biological functions such as chromatin repackaging, genomic imprinting, early embryonic development (Das et al., 2013), and post-fertilization events (Prakash et al., 2021). The spRNA profile also differed between low-motile and high-motile spermatozoa (Lambard et al., 2004) and between fertile and infertile men (Wang et al., 2004; Ostermeier et al., 2005). Yatsenko and co-workers detected abnormal *UBE2B* (Yatsenko et al., 2013), *ZPBP1* (Yatsenko et al., 2012) and *KLHL10* (Yatsenko et al., 2006) genes in spRNA and associated it with impaired fertility. Wu et al. (Wu et al., 2012) reported that miR-19b and miR-let7a could serve as specific and sensitive biomarkers for spermatogenic status in idiopathic infertile males with oligozoospermia and non-obstructive azoospermia. Altered miRNAs expression has also been found in the testis with non-obstructive azoospermia (Lian et al., 2009). Thus, the expression level of spRNAs and seminal RNAs may have potential use in studying the post-spermatogenesis events and detecting defects in sperm function (Prakash et al., 2021). A layout of whole transcriptome studies in major livestock species is presented in Figure 1.

### Coding RNAs in Spermatozoa of Livestock

The spRNA population has been described in various farm animals such as cattle, horses, and pigs (Card et al., 2013; Das et al., 2013). The coding RNAs were limited mainly to mRNA transcripts, whereas ncRNAs belonged to different types such as miRNAs, lncRNA, rRNA, tRNA, tsRNA, circRNA, etc. The presence of fragmented rRNAs (Cappallo-Obermann et al., 2011), 5,000–6,000 mRNAs, and other classes of RNAs such as tRNA, small RNA, and miRNA have also been described (Parthipan et al., 2017). Among protein-coding RNAs, Hydrolases, SP-40, Sulfated glycoprotein 2, Calmegin, Heat shock proteins (HSPs) were highly abundant. At the same time, non-coding RNA (ncRNA) mainly included miRNAs and siRNAs whose targets were signaling pathways such as the Wnt signaling pathway that is known to be regulators of early embryonic development and differentiation (Peifer, 2000).

### Coding spRNAs in Cattle

The whole transcriptome profiling of bulls’ spermatozoa revealed a wide range of potential transcripts (Table 1) that regulate DNA packaging, cytoskeletal organization, acrosomal reactions (e.g., *PLCB1, YWHAZ*) (Selvaraju et al., 2017), oocyte activation, embryogenesis, placental development (e.g., *PAG5, PAG7,* and *PAG10*), embryonic morphogenesis, microtubule function (e.g., KIF5C and KCNJ6), mitochondrial function (e.g., *COX5A* and *COXI1*), calcium signaling (e.g., *PLCZ1* and *PLCB1*), and centrosome organization (e.g., *MAP7, MYH9, PLK1S1, PRKCZ*, and *PTK2*). Bovine cDNA microarray analysis of spermatozoa from different segments of epididymis further revealed segmental differences in the transcriptome. Among the differentially expressed genes (DEGs), the top 10 DEGs were related to reproducti*ve* function (*ADAM28, AKAP4, CTCFL, FAM161A, ODF1, SMCP, SORD, SPATA3, SPATA18*, and *TCP11*) and five DEGs (*DEAD, CYST11, DEFB119*, DEFB124, and *MX1*) were related to the immune response and cellular defense (Légaré et al., 2017). Notably, most up-regulated transcripts of the caput epididymis are known to regulate spermatogenesis and sperm morphology and included *AKAP4, ODF1 CTCFL, SMCP, SPATA3, SPZ1*, and *SPATA18*. These genes in spermatozoa have been associated with sub-fertility of bulls with incomplete spermiogenesis (Hermo et al., 2010). The sperm transcripts related to sperm maturation were *ADAM28, CRISP2, CST11, LCN9S*, and *TCP11*. On the other hand, dysregulated immune defense-related genes were *CATGL4* and *GSTA2* in the caput epididymis; *GPX5, MX1*, and *DEFB124* in the corpus epididymis and; *DEFB7* and *DEFB119* in the cauda epididymis (Selvaraju et al., 2017).

**TABLE 1:** Important protein coding transcripts reported in cattle spermatozoa by transcriptome analysis.

Species	Study type	Groups	Transcripts (Gene symbol)	Function	References
**Cattle**	RNA-Seq	Whole transcriptome	*ZBTB20*	Spermatogenesis	Raval et al. (2019)
	RNA-Seq	Whole transcriptome	*YWHAZ*	Spermatogenesis and acrosomal reactions	Selvaraju et al. (2017)
		*KIF5C* and *KCNJ6*	Microtubule function		
			*TNP1, RAD21, UBE2B* and *RAN*	Spermatid development	
			*TEKT1*	Acrosome and on flagella and could play a key role during fertilization	
			*CAPZA3*	Spermatozoa capacitation and spermatozoa egg fusion	
			*MAP7, PTK2, PLK1S1, MYH9* and *PRKCZ*	Spermatogenesis and sperm function	
			*PLCZ1* and *PLCB1*	Calcium signalling and the spermatozoon-induced activation of an oocyte leading to its final maturation	
			*ADAM1B, ADAM2* and *ADAM32*	Spermatozoal motility	
			*ZP3* and *FOXG1*	Embryogenesis-associated transcript	
			*PAG5, PAG7* and *PAG10*	Implantation and placentation	
	RNA-Seq	High vs Low fertile	*PFN1*	Oocyte maturation, fertilization, embryo development, spermatogenesis	Prakash et al. (2021)
			*PRKCA 1*	Acrosome formation	
			*ODF1, BCL2L11, PRM2, TNP2, ODF2, SPEM1,* and *MEA1*	Spermatogenesis	
			*YBX1, UBE2B, BCL2L11, MYH10,* and *RBBP6*	*In-vitro* embryonic development	

Various intact rRNA (e.g., *RPL23, RPL27A*, and *RPS18*) and degraded rRNAs (*RPL6, RPL36AL*, and *RPL37*) have also been reported in bull spermatozoa (Montjean et al., 2012), which might suggest their potential role in spermatogenesis. The comparative RNA profiles of semen from high- and low-fertility bulls using microarray revealed 415 DEGs (out of 24,000 analyzed genes) with a significant number of fertility-associated markers (Feugang et al., 2010). The study showed higher expression of membrane and extracellular matrix genes in spermatozoa from high-fertility bulls, while transcripts of transcriptional and translational factors were lower in the low-fertility bulls. Increased expression of *CSN2* and *PRM1* in spermatozoa of high-fertility bull and lower expression of *CD36* in low -fertility bull were suggested as possible fertility markers (Feugang et al., 2010).

The role of coding spRNA transcripts was also indicated in the motility of sperm flagellum (*AKAP*) and DNA packaging (*PRM2*) (Singh et al., 2019). Differences in spRNA transcripts among high- and low-fertility dairy bulls have been reported with 805 unique transcripts in high-fertility and 2,944 unique transcripts in the low-fertility bulls (Card et al., 2017). Expression of cytochrome oxidase subunit (*COX7C*) was negatively correlates with male fertility (Card et al., 2017). In another study, a combination of five genes (*AK1, ITGB5, TIMP*, *SNRPN2*, and *PLCz1*) accounted for 97.4% of the variation of conception rate from frozen-thawed Holstein bulls, which was indicative of the sire fertility index (Kasimanickam et al., 2012). The upregulated sperm transcripts (e.g., *RPL3, PABPC1, TPT1, RPL14, RPS8, PFN1, DDX39B,* and *CD74*) in low-fertile crossbred bulls annotated to biological functions like apoptosis, cellular differentiation, apoptosis of germ cells, spermatogenesis, fertilization, and early embryo development (Arcuri et al., 2004; Rawe et al., 2006; Selvaraju et al., 2018) whereas downregulated sperm transcripts (e.g., *RUNDC3A, LYRM4*, *FAM71F1, ZFN706, PICK1, LUZP1, ANKRD9*, and *EPOP*) were found to be modulating the cytoskeletal organization and acrosome formation (Selvaraju et al., 2017). The *TSSK6, C12H13orf46*, *FABP3*, and *IQCF1* genes were the top spRNA transcripts that were unique to high-fertility bull spermatozoa and were associated with biological functions of protein phosphorylation, sperm chromatin condensation during spermiogenesis, sperm motility, acrosome reaction, and gamete fusion during fertilization (Bissonnette et al., 2009; Sosnik et al., 2009; Fang et al., 2015; Selvaraju et al., 2018). The upregulated sperm transcripts that were unique to the low-fertility bulls included ribosomal proteins and thymosin beta 10 (*TMSB10*), which are involved in sperm capacitation, fertilization, and cellular remodeling during trophoblast adhesion (Cammas et al., 2005; Selvaraju et al., 2017). These studies suggest an association between coding spRNAs in bulls sperm and their fertility.

### Coding spRNAs in Pigs

The significance of spRNA functions has also been recognized in pigs (Table 2). Using RNA-Seq, several genes linked to spermatogenesis (e.g., *FGF-14* and *BAMBI*), energy metabolism (e.g., ND6 and ACADM), protein phosphorylation (e.g., *PTPRU and PTPN2*), autophagy (e.g., RAB33B), inflammation, and apoptosis (e.g., EAF2, FOS, ITGAL, NFATC3, and ZDHHC14) have been reported to be significantly up-regulated in poor freezable ejaculates of boar semen (Fraser and Adeoya-Osiguwa, 2001). Thus, the spRNA population may serve as a molecular signature or marker of sperm freezability. Comparison of spRNAs in fresh vs. frozen-thawed boar sperm resulted differential expression of 567 protein coding mRNA and 135 non-coding miRNA (Card et al., 2017; Dai et al., 2019). The KEGG pathway analysis of DEGs revealed several signaling pathways governing the spermatogenesis process, such as chemokines signaling, PI3K-AKT, cGMP-PKG signaling, JAK-STAT signaling, calcium signaling, TNF signaling, MAPK, calcium signaling, NF-kappa B signaling, and AMPK signaling pathways. Similarly, microarray analysis of spRNA population in differentially fertile pigs revealed significant involvement of major signaling pathways associated with JAK2 and STAT3 pathways, cytokine receptor activity, activation of B-and T-lymphocytes (Yang et al., 2009). High fertile groups were also marked with downregulation of cell adhesion and proliferation genes (e.g., *CDH10, CDSN, ITGB8, ANGPTL1,* and *CTNNA3*) and upregulation of genes associated with the cellular component organization (e.g., *KRI1* and *ZNHIT6*), endocytic receptor activity (e.g., *CXCL16*), membrane channels (e.g., *KCNA3, KCNIP3, KCNH4,* and *KCTD9*) and translation regulator activity (e.g., *CPEB3*). Transcripts regulating sperm motility during *in vitro* capacitation, such as *CATSPERG* (CatSper channel auxiliary subunit gamma) was upregulated, and *CATSPERB* (CatSper channel auxiliary subunit beta) was down-regulated in high-fertility pigs. The DEGs annotating to zinc finger nucleases (ZFNs) such as *LOC100739821, ZNRF4*, *PLAGL2, ZFN25*, and *ZDHHC7* were primarily upregulated in high-fertility boars (Alvarez-Rodriguez et al., 2020). Some of the ZFN transcripts (e.g., *ZFN283, FEZF2*, and *GLI1*) were downregulated in high fertile groups and were involved with sonic hedgehog signaling pathway (Kroft et al., 2001). Apart from this, transcripts of *NFYA, TCF21, MBTPS2, MTF2, IRF3,* and *ISG20L2* were significantly overexpressed, and *UBTFL1, ADAM7, ADAM29, IL23R, IFN-DELTA-4, IFNK*, and *IFIT3* were under-expressed in high fertile boars (Turner et al., 2006; Wei et al., 2011).

**TABLE 2:** Important protein coding transcripts reported in pig spermatozoa by transcriptome analysis.

Species	Study type	Groups	Transcripts (Gene symbol)	Function	References
**Pigs**	cDNA-library	Ejaculated spermatozoa	*Hsp70.2, SSFA2, SESN1*	Embryogenesis	Yang et al. (2009)
	RNA-Seq	Capacitated sperm	*MAPK1, PGK1, PPM1B,* and *PGAM1*	Capacitation	Li et al. (2018)
	RNA-Seq	Fresh vs frozen semen	*VEGFA*	Self-renewal and maintenance of male spermatogonial stem cells, activate the AKT signalling, improve sperm motility	Dai et al. (2019)
			*DNMT3A, DNMT3B, JHDM2A, KAT8,* and *PRM1*	Epigenetic regulation	
			*PLCZ1* and *CRISP2*	Calcium ion pump, sperm–egg interaction, regulation of sperm intake, and fertilization process	
			*MTHFR, PAX8, IGF2, LIT1,* and *SNRPN*	Epigenetic regulation	
	Microarray	High fertile vs Low fertile	*AKAPs*	Capacitation, motility	Alvarez-Rodriguez et al. (2020)
			*CATSPER*	Mammalian fertilization by Calcium signalling	
			Zinc finger proteins	Transcriptional regulation, ubiquitin-mediated protein degradation, signal transduction, actin targeting, DNA repair and cell migration	
			Matrix metallo-proteases	Activation of TNF-alfa and generation of Epidermal Growth Factor Receptor	

The spRNAs enriched in cell-cell adhesion, endometrial epithelial cell receptivity, lipid and glucose metabolism, inflammation, autophagy, matrix metalloproteases, mitochondrial apoptosis, and immune-related signaling pathways have also been reported (Alvarez-Rodriguez et al., 2020). The EST library of ejaculated spermatozoa from Landrace pig deciphered some of the putative homologs of transcripts regulating embryogenesis (e.g., *HSP70.2*, *SSFA2*, and *SESN1*). These transcripts were also marked as potential regulators of spermiogenesis, oocyte fertilization, cellular growth, and cleavage (Yang et al., 2009). Seasonal variation in RNA-seq profiling of spRNAs was also reported in pigs (Yang et al., 2010) and included transcripts having functional similarity with those previously reported in cattle (Selvaraju et al., 2017). The mRNA transcripts regulating spermatogenesis (e.g., *ODF2* and *SPATA18*), nuclear genome structure (e.g., *PRM1*, *OAZ3*, *HSPB9*, and *NDUFS4*), mitochondrial function (*e.g., COX1ATP8*), and fertilization (e.g., *HSPA1L* and *PRSS37*) were observed to be associated with compaction of sperm chromatin, energy metabolism, oxidative stress, apoptosis, and early embryo development (Sendler et al., 2013; Sakurai et al., 2016; Selvaraju et al., 2017). It was also suggested that the *TSARG1* transcript in spermatozoa might be involved in inhibiting apoptosis (Yang et al., 2005). The study reported that the expression level of *TSARG1* and testis-specific kinase 1 (*TESK1*), which are members of the DnaJ-like protein family and serine/threonine kinases, respectively, were significantly higher in the sperm pools collected in winter than in summer. The transcriptome analysis of pig testis and epididymis revealed several transcripts associated with different regions of the epididymis (Guyonnet et al., 2009). Several lipocalins were observed in different epididymal segments such as *LCN6, LCN8, LCN9, LCN10, PTGDS,* but their role is not yet evident in response to epididymal functionalities.

### Coding spRNAs in Stallion

In order to establish the significance of spRNAs as potential biomarkers of stallion fertility, global transcriptome profiling of semen has been explored (Das et al., 2013; Suliman et al., 2018). The abundance of spermatozoal mRNAs (e.g., *ADIPO, AK1, CRISP2, DOPPEL, ITGB5, NGF, PEBP1, PLCZ1,* and *TIMP2*) were found to be positively correlated with fertility, while mRNA transcripts *of CCT8* and *PRM2* were found to be negatively correlated (Kasimanickam et al., 2012). The stallion sperm transcripts were predicted to regulate critical biological functions, *viz.*, the integrity of plasma membrane, RNA processing, transcription regulation, mitochondrial ribosomal protein, ion binding, chemokine receptor DNA packaging, protein folding, cytoskeleton, GTPase activator, chromatin assembly complex, and protein transport (Suliman et al., 2018). The cysteine-rich secretory protein (CRISP), found in seminal plasma proteins, was involved in gamete fusion (Töpfer-Petersen et al., 2005). Some ribosomal binding proteins (e.g., *GRTH*, *SAM68*, *MSY2*, and *DAZAP1*) were also observed that are known to promote the translation of mRNAs in germ cells. The latter may thus, suggest the importance of spRNAs in regulating protein translation during spermatogenesis (Bettegowda and Wilkinson, 2010). Comparative transcriptomics of stallion sperm identified 149 DE miRNAs between dense and less-dense spermatozoa (Ing et al., 2020) wherein *BC O 1, GLRA4, OTOL1, PRM1, SCP2D1*, and *SPATA31D1* were highly expressed in dense spermatozoa compared to those of less-dense spermatozoa. This study also found that expression of *PRM1* transcripts was significantly higher in morphologically normal spermatozoa from sub-fertile stallions and in spermatozoa with abnormal morphology (Paradowska-Dogan et al., 2014). The association of protamines expression levels in frozen-thawed semen had implications in stallion fertilization (Kadivar et al., 2020).

### Non-Coding RNAs in Spermatozoa of Livestock

Two groups of ncRNAs are the short and long ncRNAs (Watson et al., 2019). Transcripts <200 nucleotides are the small ncRNA (sncRNA) that include piwi-interacting RNA (piRNAs), small interfering RNA (siRNAs), miRNA, rRNA, tRNA, snoRNAs, and small nuclear RNA (snRNAs) (Grivna et al., 2006). Transcripts >200 nucleotides are generally categorized as long ncRNA (lncRNA) (Fort et al., 2021). Deep sequencing of cattle spRNA revealed that sncRNA population comprises the most abundant rRNA followed by piRNAs, miRNAs, and tRNA fragments (tsRNA), contradictory to sperm-based data where tRNAs represented most of the reads (Sellem et al., 2020). Microarray analysis of spRNAs in humans revealed that spRNA contains an array of ncRNAs that included miRNAs and siRNAs. Interestingly, 68 of these siRNAs had protein targets which were regulators of development and differentiation in *C. elegans* (Martins and Krawetz, 2005). The function of these sperm-derived miRNA was further demonstrated by (Amanai et al., 2006). Using anti-miRNA microinjection to oocytes, (Amanai et al., 2006), showed that spRNA has a limited function during early embryonic development in pigs. Nevertheless, direct proof of the function of these RNAs is still elusive. The presence of ncRNAs in livestock has been associated with spermatogenesis, fertilization, early embryogenesis (Prakash et al., 2020), and epigenetic modification of the sperm genome (Jodar et al., 2013; Prakash et al., 2020).

### Spermatozoal miRNA

The miRNAs are short single-stranded RNAs of ∼18–22 nucleotide length that are found abundantly in mammals and are known to post-transcriptionally regulate the function of mRNAs through inhibition or suppression of translation or degradation of mRNA themselves. The significance of miRNAs in male reproduction has been documented in the maintenance of self-renewal and differentiation of SSCs into spermatozoa as well as during the spermiogenesis process (Chen X. et al., 2017). We previously showed that the let-7 family of miRNA is involved in regulating germ cell proliferation and may be used as a marker of male germ cells (Jung et al., 2010) in addition to imprinted miRNAs (Shin et al., 2011). Similarly, miR-21, miR-34c, miR-183, miR-465a, etc., were also found to be vital for SSC self-renewal through regulation of *Etv5, Cdnd1, and Stat3* expression in mice (Niu et al., 2011; He et al., 2013). On the other hand, miR-224 and miR-322 regulate the Wnt signaling pathway for self-renewal of mice SSCs by increasing the expression of *Gfrα1, Plzf,* and *Rassf8* (Cui et al., 2016; Wang Y. et al., 2019) whereas miR-100 and miR-10b promoted the proliferation of SSCs through *Stat3* and *Klf4*, respectively (Huang et al., 2017; Li et al., 2017). The miRNAs were also shown to play crucial roles in the differentiation of mice SSCs and spermatogenesis by targeting the *Dmrt1* (Cui et al., 2016) and through the retinoic acid pathway (Bellvé et al., 1977).

The complexity of the miRNA population has also been seen in spermatozoa of several mammalian species, including cattle, pigs, stallion, mice, and humans (Capra et al., 2017; Castillo et al., 2018; Gòdia et al., 2018). However, the precise functions of these sperm miRNAs in spermatogenesis or post-fertilization events are yet to be established. Spermatozoal miRNAs are believed to play essential roles during fertilization of oocytes and early embryonic development (Castillo et al., 2018). They may also affect the abundance and epigenetic status of maternal mRNAs in oocytes and zygotes (Capra et al., 2017).

### Spermatozoal miRNAs in Cattle

Spermatozoal miRNAs have been reported in healthy bulls and those differeing in their spermatozoal motility or fertility (Table 3). The whole miRNA profiling of bull spermatozoa through deep sequencing identified 2022 miRNAs and included a large number of isomiRs across different species (Turri et al., 2021). RNA-seq analysis of high fertility and low fertility bulls reported nine DE miRNAs (miR-2285n, miR-378, miR-423-3p, miR-19, miR-2904, miR-378c, miR-431, miR-486, and miR-2478) in which miR-2285n, miR-378, and miR-486 were observed to have altered expression levels between low and high motile sperm. The highest expression of miR-2285n and miR-486 was seen in low motile spermatozoa and the semen samples from low fertility bulls. The miR-486 plays a crutial role in regulating stemness and cell proliferation of SSCs. The miR-378 targets mRNAs involved in metabolism and sperm motility (Liu et al., 2019). Differential expression of spermatozoal miRNAs was also observed upon microarray analysis of spermatozoal miRNA in low vs. high motility spermatozoal and low vs. high fertility bull (Sellem et al., 2020; Keles et al., 2021).

**TABLE 3:** Important non-coding microRNAs reported in cattle spermatozoa by transcriptome analysis.

Species	Study type	Groups	MicroRNAs	Function	References
**Bovine**	cDNA library	High fertile vs Low fertile	miR-34b/c	Sperm chromatin condensation	Lian et al. (2021)
			miR-17-92 and miR-106b-25	Spermatogenesis	
			miR-10a-5p	Motility	
	MicroRNA microarray	High and low fertile	miR-3155, miR-8197, miR-6727, miR-11796, miR-14189, miR-6125, and miR-13659	Sperm fertilization	Govindaraju et al. (2012)
	RNA-Seq	Semen sample	miR-365-2	Oocyte fertilization and cleavage	Selvaraju et al. (2017)
	RNA-Seq	Small RNA sequencing	bta-miR-103, bta-miR- 30b-5p, bta-miR-17-5p, bta-miR-106b, bta-miR-142-3p, bta-miR-34b, bta-miR-18a, bta-miR-34c, bta-miR-455-5p, bta-miR-10b, bta-miR-99b, bta-miR-1246, bta-miR-99a-5p, bta-miR-1388-5p	Motility	Capra et al. (2017)

The miRNA profiling of bull sperm using microarray presented an abundant quantity of miRNA along with DE miRNA target genes from high vs. low fertility groups (Dai et al., 2019) suggests the roles of miRNA in regulatory mechanisms. Seven important miRNAs (hsa-aga-3155, 8197, 6,125, 6,727, 11,796, 13,659 and 14,189) were DE (using human microarray probe set) and validated by qRT-PCR. The target genes of these miRNAs included *AQP7P1, CHN2-EPHA1- EFNA2, DNM2, IFT80, TOB2, CHN2, CLUL1, BC035897, BTBD2,* possibly regulating gametogenesis, acrosome integrity, and fertilization. An interactome model of miRNA target genes has also been proposed to identify the master regulator of a set of genes with a single miRNA molecule (Govindaraju et al., 2012). The miR-26a and miR-455-5p were significantly up-regulated in highly motile spermatozoa, whereas levels of miR-10a and miR-1 were significantly down-regulated. The miR-26a also participates in PTEN and PI3K/AKT signaling pathways, affecting sperm viability and motility (Dai et al., 2019). In another study, miR-34b-3p and miR-100-5p were also significantly over-expressed in spermatozoa of high fertility bull compared to those of low fertility bull (Keles et al., 2021). The miRNAs, previously known to be expressed testis, were also detected in bull spermatozoa and included miR-34b/c miRNA cluster, which plays vital roles in chromatin condensation during spermiogenesis (Capra et al., 2017). Thus, miRNA profile could be a useful indicator for high fertility spermatozoa.

The miRNA profiling has also found its application in assessing the success of semen cryopreservation protocols. Increased expression of miR-34c was seen in the highly motile cryopreserved bull spermatozoa purified through Percoll density gradient centrifugation. The miRNA profiling of differentially motile cryopreserved spermatozoa revealed that spermatozoal miRNAs target STAT3, PI3K/AKT, and PTEN signaling pathways that play essential roles during mammalian spermatogenesis, mitochondrial membrane potential, sperm maturation, and fertilization. PTEN, the target of multiple miRNAs (e.g., miR-17-5p, miR-26a-5p, and miR-486-5p), inhibits AKT signaling and activates RAF1/ERK signaling to initiate sperm maturation. Two miRNA *viz.* miR-122 and miR-184 were upregulated in low motile fractions of semen, which targets the AKT signaling pathway to cause apoptosis. In addition, miR-17-5p and miR-20a- 5p, which were found to be under-expressed in the low motile fraction of semen, targets PTEN and STAT signaling pathways to trigger apoptosis (Capra et al., 2017).

The spermatozoal miRNA have also been shown to differ in gonadotoxicity, heat stress, and impaired spermatogenesis. Differentially expressed miRNAs have been reported between normal and abnormal spermatozoa of bulls affected by Fescue toxicosis (Stowe et al., 2014). An increased expression of let-7a and miR-22 was shown in spermatozoa with abnormal morphology due to toxicosis. Further, among the most abundant miRNAs, the let-7 family and miRNA-146 were found to regulate the differentiation of spermatogonia and impaired blastocyst implantation in mice whereas, miRNA-16 is known to coordinate cell-cycle (Zhang et al., 2012). A recent study suggests miRNA (e.g., miR-126-5p) accumulates during the final stage of spermatogenesis, and epidydimal transit and their expression is affected during heat stress, which might explain reduced fertility in heat-stressed bulls due to impaired spermatogenesis and maturation (Dos Santos da Silva et al., 2021). The miR-17-92 and miR-106b-25 are two miRNA clusters whose target genes were reported to be essential for the progression of spermatogenesis (Tong et al., 2012). It was reported that spermatozoa that exhibited normal morphology had mutant miR-92a, implying that miR-92a might affect male fertility. The miR-10a and miR-9-5p have been frequently reported in cattle and pig spermatozoa. Both of these miRNAs were observed to be upregulated in cryopreserved spermatozoa. On the other hand, miR-10a-5p was found to be overexpressed in low motility spermatozoa and was associated with spermatogenesis and DNA repair capacity.

### Spermatozoal miRNAs in Pig

The miRNAome of boar spermatozoa has been described in several studies (Table 4) and may vary with season (Varona et al., 2019). The miR- 34c, miR-191, miR-30d, miR-10b, and let-7a were the most abundant spermatozoal miRNAs associated with spermatogenesis in pigs. The differential expression of several miRNAs is seen in low vs. high fertility boars, low vs. high motility, and fresh vs. cryopreserved spermatozoa. The small RNA libraries of fresh and frozen-thawed boar spermatozoa revealed several potential cell signaling pathways governed by spermatozoal miRNAs (Dai et al., 2019). For example, miR-17-5p, miR-20a-5p, miR-26a-5p, miR-122-5p, miR-184, and miR-486-5p were involved in regulating PTEN, PI3K/AKT, and STAT signaling pathways that influence sperm motility, viability, and apoptosis in frozen-thawed spermatozoa (Zhang Y. et al., 2017) The increased expression of miRNAs such as let-7a, 7d, 7e, miR-22, let-7d, and let-7e were seen in frozen-thawed spermatozoa with morphological abnormalities or low motility. The cryopreservation of boar sperm also led to reduced expression of let-7c, miR-22, miR-26a, miR-186, and miR-450b-5p in frozen-thawed boar spermatozoa (Capra et al., 2017). The differential expression of these spermatozoal miRNAs may, thus, have implications in sperm fertility as they are associated with motility and apoptosis in frozen-thawed boar spermatozoa.

**TABLE 4:** Important non-coding microRNAs reported in pig spermatozoa by transcriptome analysis.

Species	Study type	Groups	MicroRNAs	Function	References
**Pigs**	qRT-PCR	Ejaculated spermatozoa	let-7a, -7d, and -7e	Spermatogenesis	Curry et al. (2011)
			miR-22	Sperm structure	
		Epididymal and ejaculated sperm	let-7a and miR-92a	Calcium and camp signalling	Chang et al. (2016)
			ssc-let-7a, ssc-let-7d, ssc-let-7e and ssc-miR-98 regulate	Sperm apoptosis	
			miR-19 and miR-26a	AKT/PKB signalling pathway	
			miR-224, miR-19b,miR-504 and miR-676	Cell apoptosis and cell proliferation	
	RNA-Seq	Fresh vs frozen spermatozoa	miR-26a, and miR-455-5p	PTEN, and PI3K/AKT signalling pathway	Dai et al. (2019)
	RNA-Seq	Capacitated sperm	miR-127, miR-1343, miR-151-3p	Calcium signalling pathway and MAPK signalling pathway	Li et al. (2018)
	RNA-Seq	Fresh vs frozen semen	miR-17-5p, miR-26a-5p, miR-486-5p, miR-122-5p, miR-184, and miR-20a-5p	Regulate PTEN, PI3K/AKT, and STAT signalling influence motility, viability, and sperm apoptosis	Dai et al. (2019)
			let-7a, -7d, -7e, miR-22, let-7d, and let-7e	Sperm motility	
			miR-3155, miR-8197, miR-6727, miR-11796, miR-14189, miR-6125, and miR-13659	Bovine sperm fertilization	
			miRNAs, miR-26a, and miR-455-5p	Sperm motility	
			miR-10a and miR-1 were	Sperm motility	
	Microarray	High fertile vs Low fertile	miR-615	Capacitation	Alvarez-Rodriguez et al. (2020)
			miR-221	Wnt2, BDNF, CREB-related genes,PI3K-Akt and the estrogen signalling pathway	

The miRNA microarray profile of high and low fertile boars found 326 pig-specific miRNAs (Alvarez-Rodriguez et al., 2020), among which miR-1285 was found to be related to sperm production, promoting AMPK phosphorylation and regulating oxidative stress (Jiao et al., 2015). Interestingly, miR-15/miR-16 was the most abundant miRNA and is known to suppress the TGF-β signaling pathway (Ayaz and Dinç, 2018) and enhance spermatogonial proliferation and spermatogenesis in gonadotoxic patients (Moraveji et al., 2019). The mRNA targets of these miRNA are involved in various essential cellular functions such as lipid metabolism (e.g., miR-4332), cell proliferation, and apoptosis (e.g., miR-671-5p, mir-425-5p) (Qiu et al., 2018). Moreover, miR-425 and IL-23 were downregulated in high fertile bulls, whereas its receptor (*IL-23R*) was down-regulated in high-fertility boars (Lu et al., 2019). The overexpressed miRNAs in high fertile boars included miR-191, miR-42, which play significant role in regulating the motility of spermatozoa inside the female reproductive tract (Zhang et al., 2018). It has also been indicated as a key activator of the NF-κB signaling pathway, the regulator of estrogen receptors, and an inhibitor of the Wnt pathway (Alvarez-Rodriguez et al., 2020). Two down-regulated miRNAs, *viz.* miR-615, miR-221 are known to act as potential targets for EGFR and regulate acrosome reaction (Michailov et al., 2014) and PI3K-AKT and the estrogen signaling pathways (Alvarez-Rodriguez et al., 2019).

The pioneer report on sperm capacitation-specific miRNA profiling in boar was elucidated by (Li et al., 2018). This study identified DE miRNAs in fresh non-capacitated and capacitated spermatozoa. Some of the upregulated miRNAs in capacitated spermatozoa included, miR-148a-3p, miR-151-3p, miR-425-5p, miR-132, miR-451, miR7136-5p, miR-489, miR-1343, miR-1306-3p and fresh spermatozoa were enriched for miR-378b-3p, miR493-5p, miR-133a-3p, miR-362, and miR-214. Pathway analysis of miRNA targets revealed their biological significance in PI3K-AKT, MAPK, cAMP-PKA, and calcium signaling pathways, which are considered necessary for protein tyrosine phosphorylation and sperm capacitation. These miRNA targets also play a significant role in cell proliferation, differentiation, sperm motility, hyper-activation, and acrosome reaction, primarily *via* the ERK (Ras/Raf/MEK/ERK) signaling pathway (Li et al., 2018). The cAMP-PKA signaling activates the Ca^2+^ channel and is considered a crucial step for sperm capacitation. The miR-134 was up-regulated in capacitated spermatozoa and targets *COL11A1* and *PDE4A* genes in PI3K-AKT and cAMP-PKA signaling pathways. Some other miRNA targets such as *AKAP3* for miR-1285, *VDAC1* and *HSPA2* for miR-127, *CATSPER4* for miR-151-3p regulate sperm motility, ATPase activity, sperm capacitation (Alvarez-rodriguez, 2017). *CABYR* and *ACRBP* regulate tyrosine phosphorylation during capacitation processes (Dong et al., 2015).

The abundance of miRNAs has also been documented in epididymal spermatozoa and seminal plasma of boar semen (Chen C. et al., 2017). A total of 221, 259, and 136 miRNAs were DE between ejaculated spermatozoa and epididymal spermatozoa; seminal plasma and epididymal spermatozoa; and seminal plasma and ejaculated spermatozoa, respectively. Further, three miRNAs *viz*. let-7a, miR-26a, and miR-10b were among the top ten most abundant miRNAs in ejaculated spermatozoa, epididymal spermatozoa, and seminal plasma. The most abundant spermatozoal miRNAs targeted mRNA transcripts of binding proteins, including metal ion binding, ATP binding, and nucleotide-binding. However, caution is to be exercised in analyzing spermatozoal miRNAs in boar spermatozoa collected at different seasons. Studies have shown that spermatozoal miRNAome may show seasonal variations in boars (Varona et al., 2019). For example, miR-34c, miR-221-3p, miR-362, miR-378, miR-106a, and miR-34c were down-regulated, whereas miR-1306-5p and miR-1249 were upregulated in boar spermatozoa collected during the winter seasons. These miRNA targets primarily regulate fatty acid metabolism and oxidative stress (Wu et al., 2017), suggesting their role during the winter season. On the other hand, miR-106b, miR-378, miR-221 were previously reported to regulate autophagy machinery in different cell lines (Zhai et al., 2013; Tan et al., 2018).

### Spermatozoal miRNAs in Stallion

A few reports have also documented the spermatozoal miRNAs in stallion spermatozoa. Direct sequencing of spermatozoal miRNAs in stallion sperm revealed 82 sperm-specific miRNAs (Das et al., 2013), out of which 68 miRNAs were previously reported in human spermatozoa (He et al., 2009). Several spermatozoal miRNAs of stallion were the same as identified in the sperm of men (Krawetz et al., 2011), boars (Curry et al., 2009), and mice (Liu et al., 2012). These spermatozoal miRNAs are noteworthy because they were absent in oocytes but were present in zygotes and were involved in regulating first cleavage division in mice (Krawetz et al., 2011; Liu et al., 2012). Three highly abundant miRNAs, *viz.* miR34B, miR34C, and miR449A, regulate early embryonic development either by direct interaction with mRNAs or *via* epigenetic mechanisms in humans (Liu et al., 2012). A total of 66 new miRNAs were reported in epididymal (cauda epididymis) spermatozoa, whose predicted pathways suggested their role in sperm motility, sperm viability, and early embryonic development (Das et al., 2013).

### Other Spermatozoal ncRNAs

#### Spermatozoal sncRNAs and piRNAs

Recent studies have documented the presence of thousands of sncRNAs in spermatozoa of several animal species. These sncRNAs comprise rRNAs, tRNAs, tsRNAs, snoRNAs, piRNA, etc. (Sellem et al., 2020) and are believed to play essential regulatory roles in spermatogenesis and determining male fertility. The piRNAs are 26–32 nucleotide-long sncRNAs that exclusively represent the germline population and are considered “guardian of germline” by transposon surveillance to protect genome integrity (Krawetz et al., 2011). At tissue-specific levels, piRNA modulates key signaling pathways both at transcription and translational levels. These sncRNAs are characterized to maintain transposon silencing and genome stability during spermatogenesis (Luo et al., 2016). The piRNAs represent the most abundant sncRNA population in human sperm (Pantano et al., 2015) associated with sperm concentration and fertilization rate. Some reports evaluated their significance as semen quality parameters for cattle spermatozoa (Capra et al., 2017).

Several growing pieces of evidences suggest that piRNAs may regulate protein-coding genes in germ cells and engage in determining sperm fertility and early embryonic development (Suh and Blelloch, 2011). Although piRNAs are generally produced from repeat-associated regions or transposons by PIWIL2/PIWIL4-directed pathways, the PIWIL1-directed pathway is the primary production pathway in mature bull spermatozoa. In pig spermatozoa, piRNAs have been annotated to sperm morphology, spermatogenesis, acrosomal reaction, sperm hyperactivation, and male fertility (Ablondi et al., 2021). Some potential coding transcripts falling within the 5 kb region of centered piRNA are *CATSPER2*, *CATSPERG*, *OAZ3*, *ODF1*, *ODF2*, *PRM1*, *TEX14*, *TSSK2*, *TSSK3*, and *TSSK6*. The DE piRNAs in boar spermatozoa regulate spermatogenesis-related cell signaling pathways such as cAMP, cGMP, MAPK, and PI3K–AKT signaling pathways (Wang et al., 2021). Thus, taken together, the involvement of piRNA in the maintenance of genomic integrity during spermatogenesis and fertility seems to be a potential molecular parameter for evaluating male fertility in livestock animals.

#### Spermatozoal lncRNAs

Preliminary reports on lncRNAs considered these classes of RNAs as noise in the RNA content of a species due to lack of any protein-coding open coding frames (ORF) (Brownmiller et al., 2020). Later on, collective evidences supported their roles in epigenetic regulation, controlling transcription, and post-transcriptional mechanism (Peris-Frau et al., 2019). The role of lncRNA on spermatogenesis has been predicted by several researchers (Wallrapp et al., 2001; Bianchi et al., 2006; Forsberg et al., 2014; Falchi et al., 2018; Peris-Frau et al., 2019; Xu X. et al., 2020), but functional studies exploring their mechanism of action and relevance to spermatozoal fertility are lacking. Many targets of spermatozoal lncRNAs have been predicted (Gao et al., 2019) and were found to be enriched in apoptosis (e.g., PI3K-AKT, p53) (Xiong et al., 2019) and capacitation-related pathways (e.g., Calcium, cAMP, and MAPK signaling) (Gao et al., 2017). In stallion, comparative transcriptomics of dense and less-dense spermatozoa revealed 1,492 bp lncRNA as the most prevalent RNA (Ing et al., 2020). Further, the expression of 159 RNAs was higher in dense spermatozoa than in less-dense spermatozoa. Importantly, the dense spermatozoa resulted in a higher pregnancy rate than those achieved by unfractionated stallion spermatozoa (Morrell et al., 2011).

#### Spermatozoal circRNAs

Circular RNAs (circRNAs) are a class of ncRNAs having a closed-loop structure formed by alternative back splicing of pre-mRNA in which the 3′-end of an exon is spliced to the 5′-end of an upstream exon (Gòdia et al., 2020). Depending on their genomic locations, they can be of exonic, intronic, and intergenic types (Zhou et al., 2019). The exonic circRNAs are preferentially located in the cytoplasm, whereas intronic and intergenic circRNAs are mainly found in the nucleus. Key circRNAs can participate in testis development or spermatogenesis (Xiong et al., 2019). CircRNAs Sry (circSry) is the first reported testicular circRNA in mice (Capel et al., 1993), whereas the first report on circRNA of spermatozoa was reported in boars (Gòdia et al., 2020). Gene Ontology of circRNA revealed their epigenetic functions such as histone modification, histone H3-K36 methylation, and chromatin organization during spermatogenesis, embryonic development. Four genes *viz. ATP6V0A2, PPA2, PAIP2*, and *PAXIP1* have been directly implicated in spermatozoal function and male fertility. Among these, the PPA2 is a pyrophosphatase enzyme located at the mitochondrial membrane and is involved in ATP production during sperm capacitation and motility (Asghari et al., 2017). On the other hand, *PAXIP1* plays crucial functions in genomic stability and chromatin condensation during spermiogenesis, and its knockout resulted in testicular atrophy and male infertility in mice (Schwab et al., 2013). The TESK2 is a protein kinase that is primarily expressed in round spermatids and is predicted to function during the early stages of spermatogenesis (Røsok et al., 1999). The SPATA19 is vital for mitochondrial function and ATP production for sperm motility and fertilization (Luo et al., 2014). Genes related to early embryonic development (e.g., *ANGPT1,* CDC73, *DHX36, IPMK, RICTOR,* etc.) have also been found to be influenced by circRNAs. Remarkably, four circRNAs host genes (*DENND1B, PTK2, SLC5A10*, and *CAMSAP1*) showed a significant correlation with the motility of spermatozoa.

The circRNAs reported in adult vs. piglet testis (Zhang et al., 2021) were involved in regulating spermatogenic events (e.g., circRNA 10979 derived from *POC1A* gene) and germ cell development (e.g., circRNA 18456 derived from the *TDRD1* gene). Due to their stability and spatiotemporal specificity, circRNAs such as circRNA 10187, circRNA 6,682, circRNA 10979, and circRNA 18456 could be used as biomarkers of boar sexual maturity. The circRNA 1774 (derived from *CDC42* gene) and circRNA 18184 (derived from *PTEN* gene) were significantly downregulated whereas circRNA 40370 (derived from the RICTOR gene) was significantly upregulated in the testis of the sexually mature boars. The circRNAs were involved in signaling pathways that regulate stem cell pluripotency (e.g., *AKT3, AVCVR2A, FGFR1, ACVR1, FZD3, SMAD4*), tight junction (*MYH15, PRKCA, AMOTL1, PPP2CB, CDC42, PTEN*), and adhesion connections (e.g., *LEF1, NECTIN3, SMAD2, AFDN*), hedgehog signaling pathways (e.g., *HHIP, GSK3B, PTCH1, SMO, RAB23*), cAMP signaling pathways (e.g., *PLD1, TIAM1, GNAI1, ADCY1*), mTOR signaling pathways (e.g., *BRAF, RICTOR, HIF1A*), and phosphatidylinositol signaling systems (e.g., *CDS1, DGKH, PLCB1, DGKK*)*.* The expression profile of circRNAs in neonatal and adult cattle testis revealed some of the potential target genes of circRNAs mainly involved in cell-to-cell junction, oocyte maturation, and TGFβ signaling pathway (Gao et al., 2018). These transcripts included *PIWIL1, DPY19L2, SLC26A8, IFT81, SMC1B, IQCG,* and *TTLL5*. The host genes were associated with spermatogenesis and included *PIWIL1, SPATA6, TGFβ2, TGFBR2, ACVR2A*, and *SMAD2*.

### RNA Population in Seminal Plasma and Their Contribution to spRNA

Seminal plasma is the secretions of accessory glands containing proteins and RNAs and has been overlooked for decades. Seminal plasma components have no role in assisted reproductive technologies (ART) and, therefore, are excluded from IVF and intracytoplasmic injection (ICSI) of ejaculated sperm. The ARTs have resulted in successful fertilization, pregnancy, and live birth of offspring in humans (Marzano et al., 2020) and animals (Morgan et al., 2020) without the use of seminal plasma. However, recent studies have found that the spRNA population may be contributed by seminal plasma via exosome. Exosomes of seminal plasma are released by epididymis and other accessory sex organs, which interact with spermatozoa to unload RNA cargo to the sperm. Therefore, analyzing the exosome of the epididymis (epididymosomes) or seminal plasma may be used as a marker of reproductive disease and male infertility [reviewed in (Vickram et al., 2021)]. Further, each ejaculation contain trillions of exosomes that aids in fertilization in two ways, 1) exosome exerts immunosuppressive effects on cells of mucosa layer and, 2) transfer of RNAs to the spermatozoa (Jodar, 2019). In humans, exosomes contained miRNAs (21.7% of the total RNA), Y-RNA, mRNAs, mature piwi-RNAs, and tRNAs, suggesting that exosome delivers regulatory signals to the spermatozoa (Vojtech et al., 2014).

In pigs, sequencing of seminal plasma-derived extracellular vesicles showed diverse small RNAs, including mRNA (25% of the total reads), tsRNA (0.01% of the total reads), miRNA, and piRNA. There were 325 miRNAs, of which 37 novel miRNAs were identified in boar (Xu Z. et al., 2020). The study identified the adverse effects of miR-21-5p on boar sperm fertility. Likewise, miRNA and piRNA have also been identified in beef bulls’ seminal plasma. Of 617 small RNA, nine miRNA were DE between high and low fertility beef bulls (Stephanie, 2016). The role of seminal plasma RNA in determining male fertility seems to be undeniable. In another study, expression of *PRM1* in seminal plasma was positively correlated with mitochondrial membrane potential, and lateral movement of sperm head was associated with expression of *BMP2*, *UBE2D3*, *TRADD*, and *CASP3* (Shilpa et al., 2017). In this study, investigators have found a positive association of high expression of nerve growth factor (NGF) in the maintenance of post-thaw integrity of membrane of bull’s spermatozoa. More studies need to be conducted in other livestock animals as well to ascertain the effectiveness of seminal plasma RNAs as markers of the reproductive performance of male animals.

### Epigenetic Control of spRNA Expression

The terminal stages of spermatogenesis are accompanied by chromatin condensation and the replacement of histones by protamines. These changes are primarily driven by transition proteins and cleavage of rRNAs by nuclease activities and lead to the progressive shutdown of transcriptional and translational activities in mature spermatozoa (Jodar, 2019). Interestingly, however, some studies have documented post-translational modifications of histones in mature spermatozoa and included acetylation (Johnson et al., 2011), ubiquitination (Wang T. et al., 2019), methylation (Johnson et al., 2011), and phosphorylation (Dada et al., 2012) that are known to be involved in regulating chromatin remodeling. Thus, some canonical structures of histone variants appear to have remained unchanged throughout the final stages of spermatogenesis to maintain a hierarchical layer of genomic organization in paternal chromatin. Indeed, studies have shown that about 15% of the paternal histone remains associated with the genome in mature spermatozoa (Ward, 2009). Initially, these segments were considered a mark of partial/incomplete transition of histone-protamine exchange, but gradually these histone segments were noted with a significant regulatory network by post-translational modifications (Barrachina et al., 2018). Notably, the histone retention in spermatozoa was not randomly positioned on chromosomes, instead distinctly localized within the nucleus (Johnson et al., 2011). It is, therefore, believed that specific regions in sperm chromatin are differentially marked with modified histones to have the potential for transcription through epigenetic regulations (Krawetz et al., 2011).

**FIGURE 2:**
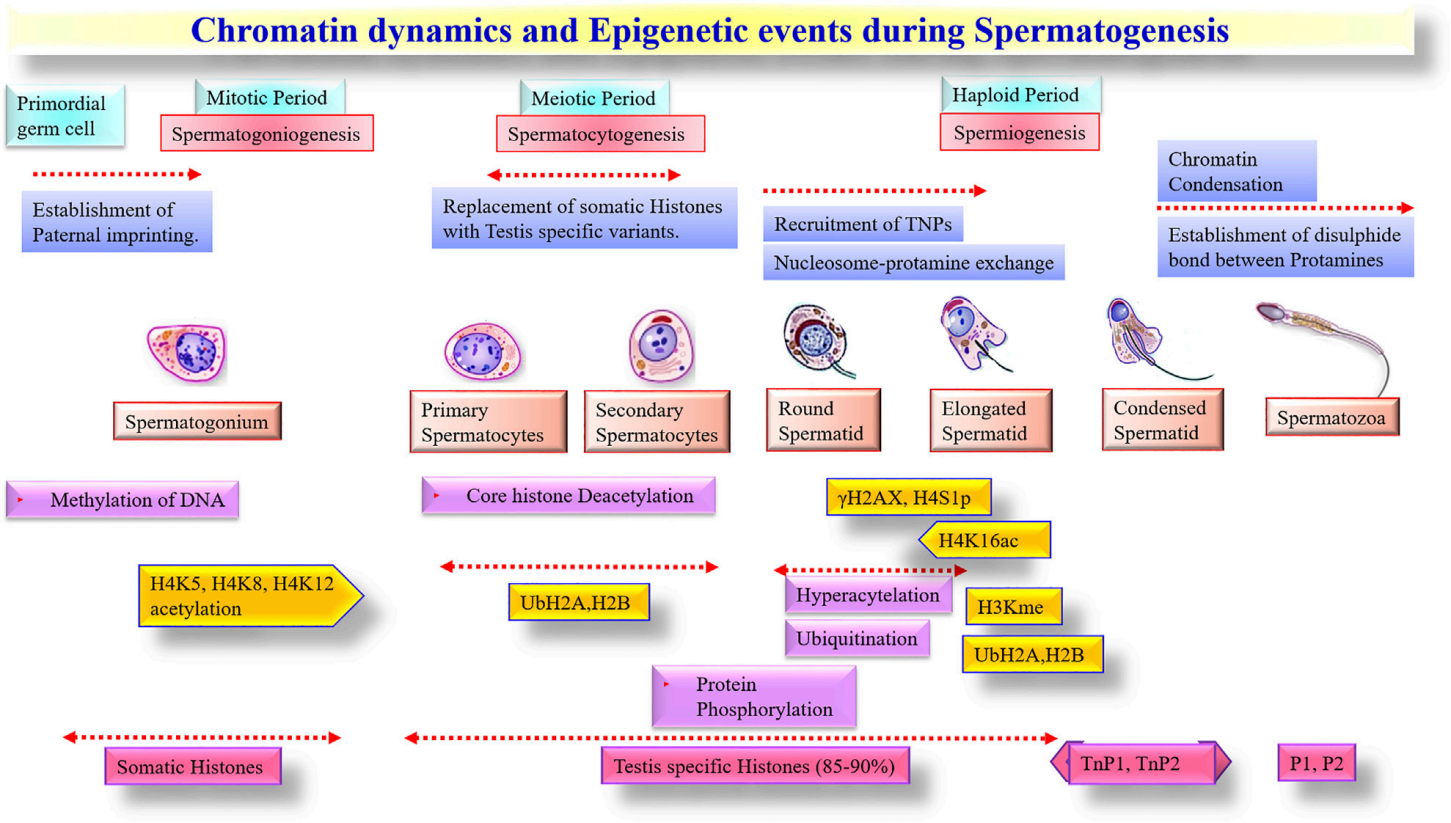
Chromatin dynamics and Epigenetic events during Spermatogenesis.

A substantial proportion of coding spRNAs detected in mature sperm are also considered as a spermatogenic leftover from transcription events during the spermatogenesis process (Jodar, 2019) and were under epigenetic control. During spermatid elongation, protamines undergo phosphorylation by serine/arginine protein-specific kinase 1 (*SRPK1*) and calcium/calmodulin-dependent protein kinase 4 (*CAMK4*), followed by rapid dephosphorylation to establish disulfide bonds between the unmasked cysteine residues of dephosphorylated protamines (Carrell et al., 2007). Histone acetylation was shown to be essential for the initiation of chromatin remodeling in spermatids (Stiavnicka et al., 2016). The hyperacetylated testicular histones were gradually replaced upon relaxation of the nucleosome complex. Testicular histone variants (*H2B and TH2B*) incorporate into spermatids with the help of transition proteins (*TNP1, TNP2*), which supersede with protamines (*PRM1, PRM2*) (Carrell et al., 2007). Histone methylation also has a significant role in the differentiation of spermatogonia, especially for H3me (H3 methylation) and H4me (H4 methylation) variants. It was reported that reduced H4me and hyperacetylation of H4 during spermatid elongation collectively mediated the histone-protamine replacement (SHIRAKATA et al., 2014). The significance of ubiquitination has also been implicated in spermatogenesis and sperm maturation to eliminate dead and defective spermatozoa and epididymal cells (Røsok et al., 1999). The stage-specific significance of epigenetic alternations during spermatogenesis is summarised (Figure 2).

### Relevance of spRNAs in Sperm Function in Livestock Species

While recent literature on Microarray and high-throughput RNAseq data have provided novel information on the repertoire of RNA population in spermatozoa, their functional validation is largely missing in the literature. Moreover, common genes and pathways across different species are yet to be fully deciphered. Nevertheless, the involvement of spRNAs in the ontology of capacitation, motility, and fertilization are apparent in various species. Among all spRNAs, protamines were the most prominent sperm-specific transcript, which is known to affect fertility in cattle (Chen et al., 2015), pigs (Gòdia et al., 2018), horses (Kadivar et al., 2020), and human (Jodar et al., 2013). Similarly, the phospholipase C (PLC)-mediated pathway was the most common pathway affected by spRNA in cattle (Selvaraju et al., 2017), pigs (Kasimanickam and Kastelic, 2016), and horses (Varner et al., 1993). The PLC is an essential regulator of intracellular Ca^2+^ oscillations that play a critical role during oocyte activation. Several studies also identified Calcium ion channels such as *CatSper* in the spRNA population, which is essential in regulating capacitation and sperm motility in livestock species as well as humans (Jan et al., 2017; Li et al., 2018; Singh et al., 2019; Nicolas et al., 2020). Besides these common sperm transcripts across various livestock species, there were flagellar specific spRNAs and sperm motility proteins such as *BSP* (de Souza et al., 2017; Pardede et al., 2020), AKAP (Gilbert et al., 2007; Chatterjee et al., 2010; Kasimanickam and Kastelic, 2016; Ing et al., 2020), *ODF* (Chen et al., 2015; Li et al., 2018; Pardede et al., 2020; Özbek et al., 2021), zinc finger nucleases (Card et al., 2013; Kasimanickam and Kastelic, 2016; Corral-Vazquez et al., 2021) and heat shock proteins. The heat shock proteins are a group of tyrosine regulatory elements that participate in hyperactivation and nitric oxide synthesis during fertilization (Feugang et al., 2010; Mohamad et al., 2018; Nicolas et al., 2020; Lian et al., 2021; Sun et al., 2021). The spRNAs such as calmegin (Guyonnet et al., 2009; Card et al., 2013), clusterin (Singh et al., 2019; Lian et al., 2021; Zhao et al., 2021), TSSK (Bissonnette et al., 2009; Li et al., 2020; Ablondi et al., 2021), and *CABYR* (Selvaraju et al., 2017) were also reported in cattle and pigs, are known to be essential for male fertility.

A few transcripts related to sperm capacitation were also reported in the spRNA population of cattle, pig, and horse spermatozoa. The important spRNAs related to sperm capacitation included CABYR (Bailey, 2010) and AKAP (Maciel et al., 2018), which regulate calcium-binding and tyrosine phosphorylation. On the other hand, *CatSper, VDAC1, and HSPA2* are involved in calcium signaling whereas*, COL11A1, PDE4A* can participate in PI3K-Akt and cAMP-PKA signaling pathways (Li et al., 2018), The *HSP70* (Mohamad et al., 2018) and *HSP90* (Jin and Yang, 2017), involved in calcium signaling, were also reported in spRNA population. Thus, it appears that calcium signaling and tyrosine phosphorylation are important events of capacitation that spRNAs may mediate. Functional studies such as gain or loss-of function research are warranted to verify these findings further.

## Conclusion

In conclusion, various types of protein-coding and non-coding spRNAs have been documented in the literature with their potential roles in regulating male reproduction and fertility. These spRNAs may be exploited *via* transcriptome analysis of spermatozoa to improve the conception rate of livestock by crossbreeding, artificial insemination, or ARTs. Microarray and RNA-seq have been extensively used as high throughput transcriptome analysis tools and have established sperm transcriptome as a subset yet distinct from testicular transcriptome with some uniquely expressed transcripts in spermatozoa. The spRNA profile, generated by microarray or RNA-seq, may serve as a molecular signature to identify semen with superior fertilizability, freezability, and fertility. Alternatively, exosome analysis of seminal plasma, which contributes to the spRNA population, may be used as a proxy to spRNAs profiling. It is also emphasized that analysis of spRNA population by high sensitive high throughput sequencing technologies should involve stringent quality control measures to avoid somatic cell contamination and batch-to-batch variation in spRNA isolation protocols. Further, loss- and gain-of function studies are required to validate the function of spRNAs in spermatogenesis, fertilization, and early embryonic development. Profiling of spRNA may also prove helpful in understanding the mechanism of action of genotoxic agents, drugs, capacitation agents, motility enhancers, etc. The next generation sequencing (NGS) of spRNA may find its application in semen evaluation for diagnosing idiopathic male infertility and devising newer methods for treatment.

## References

[B1] Ablondi M. Gòdia M. Rodriguez-Gil J. E. Sánchez A. Clop A. (2021). Characterisation of Sperm piRNAs and Their Correlation with Semen Quality Traits in Swine. Anim. Genet. 52, 114–120. 10.1111/age.13022 33226164

[B2] Alvarez-Rodriguez M. Atikuzzaman M. Venhoranta H. Wright D. Rodriguez-Martinez H. (2019). Expression of Immune Regulatory Genes in the Porcine Internal Genital Tract Is Differentially Triggered by Spermatozoa and Seminal Plasma. Int. J. Mol. Sci. 20. 10.3390/ijms20030513 PMC6387272 30691059

[B3] Alvarez-rodriguez M. (2017). iMedPub Journals Exogenous Individual Lecithin- Phospholipids ( Phosphatidylcholine and Phosphatidylglycerol ) Cannot Prevent the Oxidative Stress Imposed by Cryopreservation of Boar Sperm Abstract.

[B4] Alvarez-Rodriguez M. Martinez C. Wright D. Barranco I. Roca J. Rodriguez-Martinez H. (2020). The Transcriptome of Pig Spermatozoa, and its Role in Fertility. Int. J. Mol. Sci. 21, 1572. 10.3390/ijms21051572 PMC7084236 32106598

[B5] Amanai M. Brahmajosyula M. Perry A. C. F. (2006). A Restricted Role for Sperm-Borne MicroRNAs in Mammalian Fertilization. Biol. Reprod. 75, 877–884. 10.1095/biolreprod.106.056499 16943360

[B6] Arcuri F. Papa S. Carducci A. Romagnoli R. Liberatori S. Riparbelli M. G. (2004). Translationally Controlled Tumor Protein (TCTP) in the Human Prostate and Prostate Cancer Cells: Expression, Distribution, and Calcium Binding Activity. Prostate 60, 130–140. 10.1002/pros.20054 15162379

[B7] Asghari A. Marashi S.-A. Ansari-Pour N. (2017). A Sperm-specific Proteome-Scale Metabolic Network Model Identifies Non-glycolytic Genes for Energy Deficiency in Asthenozoospermia. Syst. Biol. Reprod. Med. 63, 100–112. 10.1080/19396368.2016.1263367 28085499

[B8] Ayaz L. Dinç E. (2018). Evaluation of microRNA Responses in ARPE-19 Cells against the Oxidative Stress. Cutan. Ocul. Toxicol. 37, 121–126. 10.1080/15569527.2017.1355314 28707489

[B9] Bailey J. L. (2010). Factors Regulating Sperm Capacitation. Syst. Biol. Reprod. Med. 56, 334–348. 10.3109/19396368.2010.512377 20849222

[B10] Balhorn R. (2007). The Protamine Family of Sperm Nuclear Proteins. Genome Biol. 8, 227. 10.1186/gb-2007-8-9-227 17903313 PMC2375014

[B11] Barrachina F. Soler-Ventura A. Oliva R. Jodar M. (2018). Sperm Nucleoproteins (Histones and Protamines). A. Clin. Guid. Sperm DNA Chromatin Damage, 31–51. 10.1007/978-3-319-71815-6_2

[B12] Bellvé A. R. Cavicchia J. C. Millette C. F. O’Brien D. A. Bhatnagar Y. M. Dym M. (1977). Spermatogenic Cells of the Prepuberal Mouse. Isolation and Morphological Characterization. J. Cel Biol. 74, 68–85. 10.1083/jcb.74.1.68 PMC2109873 874003

[B13] Bettegowda A. Wilkinson M. F. (2010). Transcription and post-transcriptional Regulation of Spermatogenesis. Philos. Trans. R. Soc. B Biol. Sci. 365, 1637–1651. 10.1098/rstb.2009.0196 PMC2871917 20403875

[B14] Bianchi N. O. Richard S. M. Pavicic W. (2006). Y Chromosome Instability in Testicular Cancer. Mutat. Res. 612, 172–188. 10.1016/j.mrrev.2005.12.001 16483836

[B15] Bissonnette N. Lévesque-Sergerie J. P. Thibault C. Boissonneault G. (2009). Spermatozoal Transcriptome Profiling for Bull Sperm Motility: A Potential Tool to Evaluate Semen Quality. Reproduction 138, 65–80. 10.1530/REP-08-0503 19423662

[B16] Boerke A. Dieleman S. J. Gadella B. M. (2007). A Possible Role for Sperm RNA in Early Embryo Development. Theriogenology 68, 147–155. 10.1016/j.theriogenology.2007.05.058 17583784

[B19] Brownmiller T. Juric J. A. Ivey A. D. Harvey B. M. Westemeier E. S. Winters M. T. (2020). Y Chromosome LncRNA Are Involved in Radiation Response of Male Non-small Cell Lung Cancer Cells. Cancer Res. 80, 4046–4057. 10.1158/0008-5472.CAN-19-4032 32616503 PMC7541653

[B20] Cammas L. Reinaud P. Dubois O. Bordas N. Germain G. Charpigny G. (2005). Identification of Differentially Regulated Genes during Elongation and Early Implantation in the Ovine Trophoblast Using Complementary DNA Array Screening1. Biol. Reprod. 72, 960–967. 10.1095/biolreprod.104.034801 15616222

[B21] Capel B. Swain A. Nicolis S. Hacker A. Walter M. Koopman P. (1993). Circular Transcripts of the Testis-Determining Gene Sry in Adult Mouse Testis. Cell 73, 1019–1030. 10.1016/0092-8674(93)90279-Y 7684656

[B22] Cappallo-Obermann H. Schulze W. Jastrow H. Baukloh V. Spiess A. N. (2011). Highly Purified Spermatozoal RNA Obtained by a Novel Method Indicates an Unusual 28S/18S rRNA Ratio and Suggests Impaired Ribosome Assembly. Mol. Hum. Reprod. 17, 669–678. 10.1093/molehr/gar037 21593214

[B23] Capra E. Turri F. Lazzari B. Cremonesi P. Gliozzi T. M. Fojadelli I. (2017). Small RNA Sequencing of Cryopreserved Semen from Single Bull Revealed Altered miRNAs and piRNAs Expression between High- and Low-Motile Sperm Populations. BMC Genomics 18, 14. 10.1186/s12864-016-3394-7 28052756 PMC5209821

[B24] Card C. J. Anderson E. J. Zamberlan S. Krieger K. E. Kaproth M. Sartini B. L. (2013). Cryopreserved Bovine Spermatozoal Transcript Profile as Revealed by High-Throughput Ribonucleic Acid Sequencing1. Biol. Reprod. 88, 1–9. 10.1095/biolreprod.112.103788 23303677

[B25] Card C. J. Krieger K. E. Kaproth M. Sartini B. L. (2017). Oligo-dT Selected Spermatozoal Transcript Profiles Differ Among Higher and Lower Fertility Dairy Sires. Anim. Reprod. Sci. 177, 105–123. 10.1016/j.anireprosci.2016.12.011 28081858

[B26] Carrell D. T. Emery B. R. Hammoud S. (2007). Altered Protamine Expression and Diminished Spermatogenesis: what Is the Link? Hum. Reprod. Update 13, 313–327. 10.1093/humupd/dml057 17208950

[B27] Castillo J. Jodar M. Oliva R. (2018). The Contribution of Human Sperm Proteins to the Development and Epigenome of the Preimplantation Embryo. Hum. Reprod. Update 24, 535–555. 10.1093/humupd/dmy017 29800303

[B28] Chang Y. Dai D. hui. Li Y. Zhang Y. Zhang M. Zhou G. b. (2016). Differences in the Expression of microRNAs and Their Predicted Gene Targets between Cauda Epididymal and Ejaculated Boar Sperm. Theriogenology 86, 2162–2171. 10.1016/j.theriogenology.2016.07.012 27527406

[B29] Chatterjee M. Nandi P. Ghosh S. Sen P. C. (2010). Regulation of Tyrosine Kinase Activity during Capacitation in Goat Sperm. Mol. Cel. Biochem. 336, 39–48. 10.1007/s11010-009-0261-8 19802524

[B30] Chen C. Wu H. Shen D. Wang S. Zhang L. Wang X. (2017a). Comparative Profling of Small RNAs of Pig Seminal Plasma and Ejaculated and Epididymal Sperm. Reproduction 153, 785–796. 10.1530/REP-17-0014 28314792

[B32] Chen X. Li X. Guo J. Zhang P. Zeng W. (2017b). The Roles of microRNAs in Regulation of Mammalian Spermatogenesis. J. Anim. Sci. Biotechnol. 8, 1–8. 10.1186/s40104-017-0166-4 28469844 PMC5410700

[B33] Chen X. Wang Y. Zhu H. Hao H. Zhao X. Qin T. (2015). Comparative Transcript Profiling of Gene Expression of Fresh and Frozen-Thawed Bull Sperm. Theriogenology 83, 504–511. 10.1016/j.theriogenology.2014.10.015 25459024

[B35] Corral-Vazquez C. Blanco J. Aiese Cigliano R. Sarrate Z. Rivera-Egea R. Vidal F. (2021). The RNA Content of Human Sperm Reflects Prior Events in Spermatogenesis and Potential post-fertilization Effects. Mol. Hum. Reprod. 27, 1–15. 10.1093/molehr/gaab035 33950245

[B36] Cui N. Hao G. Zhao Z. Wang F. Cao J. Yang A. (2016). MicroRNA-224 Regulates Self-Renewal of Mouse Spermatogonial Stem Cells via Targeting DMRT1. J. Cel. Mol. Med. 20, 1503–1512. 10.1111/jcmm.12838 PMC4956939 27099200

[B37] Curry E. Ellis S. E. Pratt S. L. (2009). Detection of Porcine Sperm MicroRNAs Using a Heterologous MicroRNA Microarray and Reverse Transcriptase Polymerase Chain Reaction. Mol. Reprod. Dev. 76, 218–219. 10.1002/mrd.20980 19012322

[B38] Curry E. Safranski T. J. Pratt S. L. (2011). Differential Expression of Porcine Sperm microRNAs and Their Association with Sperm Morphology and Motility. Theriogenology 76, 1532–1539. 10.1016/j.theriogenology.2011.06.025 21872314

[B39] Dada R. Kumar M. Jesudasan R. Fernández J. L. Gosálvez J. Agarwal A. (2012). Epigenetics and its Role in Male Infertility. J. Assist. Reprod. Genet. 29, 213–223. 10.1007/s10815-012-9715-0 22290605 PMC3288140

[B40] Dadoune J. P. (2009). Spermatozoal RNAs: What about Their Functions? Microsc. Res. Tech. 72, 536–551. 10.1002/jemt.20697 19283828

[B41] Dai D.-H. Qazi I. Ran M.-X. Liang K. Zhang Y. Zhang M. (2019). Exploration of miRNA and mRNA Profiles in Fresh and Frozen-Thawed Boar Sperm by Transcriptome and Small RNA Sequencing. Int. J. Mol. Sci. 20, 802. 10.3390/ijms20040802 PMC6413023 30781801

[B42] Das P. J. McCarthy F. Vishnoi M. Paria N. Gresham C. Li G. (2013). Stallion Sperm Transcriptome Comprises Functionally Coherent Coding and Regulatory RNAs as Revealed by Microarray Analysis and RNA-Seq. PLoS One 8, 1–17. 10.1371/journal.pone.0056535 PMC3569414 23409192

[B43] Das P. J. Paria N. Gustafson-Seabury A. Vishnoi M. Chaki S. P. Love C. C. (2010). Total RNA Isolation from Stallion Sperm and Testis Biopsies. Theriogenology 74, 1099–1106. 10.1016/j.theriogenology.2010.04.023 20615536

[B44] de Souza A. P. B. Schorr-Lenz Â. M. Lucca F. Cunha Bustamante-Filho I. (2017). The Epididymis and its Role on Sperm Quality and Male Fertility. Anim. Reprod. 14, 1234–1244. 10.21451/1984-3143-AR955

[B45] Dong H. T. Shi W. S. Tian Y. Cao L. P. Jin Y. (2015). Expression and Tyrosine Phosphorylation of Sp32 Regulate the Activation of the Boar Proacrosin/acrosin System. Genet. Mol. Res. 14, 2374–2383. 10.4238/2015.March.27.23 25867384

[B46] Dos Santos da Silva L. Borges Domingues W. Fagundes Barreto B. da Silveira Martins A. W. Dellagostin E. N. Komninou E. R. (2021). Capillary Electroporation Affects the Expression of miRNA-122-5p from Bull Sperm Cells. Gene 768, 145286. 10.1016/j.gene.2020.145286 33144270

[B47] Falchi L. Galleri G. Zedda M. T. Pau S. Bogliolo L. Ariu F. (2018). Liquid Storage of Ram Semen for 96 H: Effects on Kinematic Parameters, Membranes and DNA Integrity, and ROS Production. Livest. Sci. 207, 1–6. 10.1016/j.livsci.2017.11.001

[B48] Fang P. Xu W. Li D. Zhao X. Dai J. Wang Z. (2015). A Novel Acrosomal Protein, IQCF1, Involved in Sperm Capacitation and the Acrosome Reaction. Andrology 3, 332–344. 10.1111/andr.296 25380116

[B49] Feugang J. M. Rodriguez-Osorio N. Kaya A. Wang H. Page G. Ostermeier G. C. (2010). Transcriptome Analysis of Bull Spermatozoa: Implications for Male Fertility. Reprod. Biomed. Online 21, 312–324. 10.1016/j.rbmo.2010.06.022 20638337

[B50] Forsberg L. A. Rasi C. Malmqvist N. Davies H. Pasupulati S. Pakalapati G. (2014). Mosaic Loss of Chromosome Y in Peripheral Blood Is Associated with Shorter Survival and Higher Risk of Cancer. Nat. Genet. 46, 624–628. 10.1038/ng.2966 24777449 PMC5536222

[B51] Fort V. Khelifi G. Hussein S. M. I. (2021). Long Non-coding RNAs and Transposable Elements: A Functional Relationship. Biochim. Biophys. Acta - Mol. Cel Res. 1868, 118837. 10.1016/j.bbamcr.2020.118837 32882261

[B52] Foxcroft G. R. Dyck M. K. Ruiz-Sanchez A. Novak S. Dixon W. T. (2008). Identifying Useable Semen. Theriogenology 70, 1324–1336. 10.1016/j.theriogenology.2008.07.015 18775561

[B53] Fraser L. R. Adeoya-Osiguwa S. A. (2001). Fertilization Promoting Peptide — A Possible Regulator of Sperm Function *In Vivo* . Vitam. Horm. 63, 1–28. 10.1016/S0083-6729(01)63001-2 11358112

[B54] Gao F. Zhang P. Zhang H. Zhang Y. Zhang Y. Hao Q. (2017). Dysregulation of Long Noncoding RNAs in Mouse Testes and Spermatozoa after Exposure to Cadmium. Biochem. Biophys. Res. Commun. 484, 8–14. 10.1016/j.bbrc.2017.01.091 28111341

[B55] Gao Y. Li S. Lai Z. Zhou Z. Wu F. Huang Y. (2019). Analysis of Long Non-coding RNA and mRNA Expression Profiling in Immature and Mature Bovine (*Bos taurus*) Testes. Front. Genet. 10, 1–13. 10.3389/fgene.2019.00646 31333723 PMC6624472

[B56] Gao Y. Wu M. Fan Y. Li S. Lai Z. Huang Y. (2018). Identification and Characterization of Circular RNAs in Qinchuan Cattle Testis. R. Soc. Open Sci. 5. 10.1098/rsos.180413 PMC6083711 30109096

[B57] Gilbert I. Bissonnette N. Boissonneault G. Vallée M. Robert C. (2007). A Molecular Analysis of the Population of mRNA in Bovine Spermatozoa. Reproduction 133, 1073–1086. 10.1530/REP-06-0292 17636162

[B58] Gòdia M. Castelló A. Rocco M. Cabrera B. Rodríguez-Gil J. E. Balasch S. (2020). Identification of Circular RNAs in Porcine Sperm and Evaluation of Their Relation to Sperm Motility. Sci. Rep. 10, 1–11. 10.1038/s41598-020-64711-z 32409652 PMC7224279

[B59] Gòdia M. Mayer F. Q. Nafissi J. Castelló A. Rodríguez-Gil J. E. Sánchez A. (2018). A Technical Assessment of the Porcine Ejaculated Spermatozoa for a Sperm-specific RNA-Seq Analysis. Syst. Biol. Reprod. Med. 64, 291–303. 10.1080/19396368.2018.1464610 29696996

[B61] Gomes A. Q. Nolasco S. Soares H. (2013). Non-coding RNAs: Multi-Tasking Molecules in the Cell. Int. J. Mol. Sci. 14, 16010–16039. 10.3390/ijms140816010 23912238 PMC3759897

[B62] Govindaraju A. Uzun A. Robertson L. Atli M. O. Kaya A. Topper E. (2012). Dynamics of microRNAs in Bull Spermatozoa. Reprod. Biol. Endocrinol. 10, 82. 10.1186/1477-7827-10-82 22978562 PMC3488333

[B63] Griswold M. D. (2016). Spermatogenesis: The Commitment to Meiosis. Physiol. Rev. 96, 1–17. 10.1152/physrev.00013.2015 26537427 PMC4698398

[B64] Grivna S. T. Beyret E. Wang Z. Lin H. (2006). A Novel Class of Small RNAs in Mouse Spermatogenic Cells. Genes Dev. 20, 1709–1714. 10.1101/gad.1434406 16766680 PMC1522066

[B65] Guyonnet B. Marot G. Dacheux J.-L. Mercat M.-J. Schwob S. Jaffrézic F. (2009). The Adult Boar Testicular and Epididymal Transcriptomes. BMC Genomics 10, 369. 10.1186/1471-2164-10-369 19664223 PMC2738690

[B66] Hamatani T. (2012). Human Spermatozoal RNAs. Fertil. Steril. 97, 275–281. 10.1016/j.fertnstert.2011.12.035 22289287

[B67] Hayashi S. (2003). Mouse Preimplantation Embryos Developed from Oocytes Injected with Round Spermatids or Spermatozoa Have Similar but Distinct Patterns of Early Messenger RNA Expression. Biol. Reprod. 69, 1170–1176. 10.1095/biolreprod.103.016832 12773410

[B68] He Z. Jiang J. Kokkinaki M. Tang L. Zeng W. Gallicano I. (2013). MiRNA-20 and Mirna-106a Regulate Spermatogonial Stem Cell Renewal at the post-transcriptional Level via Targeting STAT3 and Ccnd1. Stem Cells 31, 2205–2217. 10.1002/stem.1474 23836497 PMC3859454

[B69] He Z. Kokkinaki M. Pant D. Gallicano G. I. Dym M. (2009). Small RNA Molecules in the Regulation of Spermatogenesis. Reproduction 137, 901–911. 10.1530/REP-08-0494 19318589

[B70] Hecht N. B. (1998). Molecular Mechanisms of Male Germ Cell Differentiation. BioEssays 20, 555–561. 10.1002/(SICI)1521-1878 9723004

[B71] Hermo L. Pelletier R.-M. Cyr D. G. Smith C. E. (2010). Surfing the Wave, Cycle, Life History, and Genes/proteins Expressed by Testicular Germ Cells. Part 2: Changes in Spermatid Organelles Associated with Development of Spermatozoa. Microsc. Res. Tech. 73, 279–319. 10.1002/jemt.20787 19941292

[B72] Huang Y.-L. Huang G.-Y. Lv J. Pan L.-N. Luo X. Shen J. (2017). miR-100 Promotes the Proliferation of Spermatogonial Stem Cells via Regulating Stat3. Mol. Reprod. Dev. 84, 693–701. 10.1002/mrd.22843 28569396

[B73] Ing N. H. Konganti K. Ghaffari N. Johnson C. D. Forrest D. W. Love C. C. (2020). Identification and Quantification of Coding and Long Non-coding RNAs in Stallion Spermatozoa Separated by Density. Andrology 8, 1409–1418. 10.1111/andr.12791 32243084

[B74] Jambor T. Jana B. Hana G. Eva T. Norbert L. (2017). “Male Reproduction: One of the Primary Targets of Bisphenol,” in Bisphenol A Exposure and Health Risks. Editors Erkekoglu P. Kocer-Gumusel B. (InTech). 10.5772/intechopen.68629

[B75] Jan S. Z. Vormer T. L. Jongejan A. Röling M. D. Silber S. J. de Rooij D. G. (2017). Unraveling Transcriptome Dynamics in Human Spermatogenesis. Dev 144, 3659–3673. 10.1242/dev.152413 PMC5675447 28935708

[B76] Jiao Z. J. Yi W. Rong Y. W. Kee J. D. Zhong W. X. (2015). MicroRNA-1285 Regulates 17β-Estradiol-Inhibited Immature Boar Sertoli Cell Proliferation via Adenosine Monophosphate-Activated Protein Kinase Activation. Endocrinology 156, 4059–4070. 10.1210/en.2014-1982 26287402

[B77] Jin S. K. Yang W. X. (2017). Factors and Pathways Involved in Capacitation: How Are They Regulated? Oncotarget 8, 3600–3627. 10.18632/oncotarget.12274 27690295 PMC5356907

[B78] Jodar M. Selvaraju S. Sendler E. Diamond M. P. Krawetz S. A. (2013). The Presence, Role and Clinical Use of Spermatozoal RNAs. Hum. Reprod. Update 19, 604–624. 10.1093/humupd/dmt031 23856356 PMC3796946

[B80] Jodar M. (2019). Sperm and Seminal Plasma RNAs: what Roles Do They Play beyond Fertilization? Reproduction 158, R113–R123. 10.1530/REP-18-0639 31063972

[B81] Johnson G. D. Lalancette C. Linnemann A. K. Leduc F. Boissonneault G. Krawetz S. A. (2011). The Sperm Nucleus: Chromatin, RNA, and the Nuclear Matrix. Reproduction 141, 21–36. 10.1530/REP-10-0322 20876223 PMC5358669

[B82] Jung Y. H. Gupta M. K. Shin J. Y. Uhm S. J. Lee H. T. (2010). MicroRNA Signature in Testes-Derived Male Germ-Line Stem Cells. MHR Basic Sci. Reprod. Med. 16, 804–810. 10.1093/molehr/gaq058 20610616

[B83] Kadivar A. Shams Esfandabadi N. Dehghani Nazhvani E. Shirazi A. Ahmadi E. (2020). Effects of Cryopreservation on Stallion Sperm Protamine Messenger RNAs. Reprod. Domest. Anim. 55, 274–282. 10.1111/rda.13615 31885108

[B84] Kasimanickam V. Kasimanickam R. Arangasamy A. Saberivand A. Stevenson J. S. Kastelic J. P. (2012). Association between mRNA Abundance of Functional Sperm Function Proteins and Fertility of Holstein Bulls. Theriogenology 78, 2007–2019. 10.1016/j.theriogenology.2012.07.016 23040061

[B85] Kasimanickam V. Kastelic J. (2016). MicroRNA in Sperm from Duroc, Landrace and Yorkshire Boars. Sci. Rep. 6, 1–11. 10.1038/srep32954 27597569 PMC5011730

[B86] Keles E. Malama E. Bozukova S. Siuda M. Wyck S. Witschi U. (2021). The Micro-RNA Content of Unsorted Cryopreserved Bovine Sperm and its Relation to the Fertility of Sperm after Sex-Sorting. BMC Genomics 22, 30. 10.1186/s12864-020-07280-9 33413071 PMC7792310

[B87] Kempisty B. Antosik P. Bukowska D. Jackowska M. Lianeri M. Jaśkowski J. M. (2008). Analysis of Selected Transcript Levels in Porcine Spermatozoa, Oocytes, Zygotes and Two-Cell Stage Embryos. Reprod. Fertil. Dev. 20, 513–518. 10.1071/rd07211 18462614

[B88] Krawetz S. A. Kruger A. Lalancette C. Tagett R. Anton E. Draghici S. (2011). A Survey of Small RNAs in Human Sperm. Hum. Reprod. 26, 3401–3412. 10.1093/humrep/der329 21989093 PMC3212879

[B89] Kroft T. L. Patterson J. Won Yoon J. Doglio L. Walterhouse D. O. Iannaccone P. M. (2001). GLI1 Localization in the Germinal Epithelial Cells Alternates between Cytoplasm and Nucleus: Upregulation in Transgenic Mice Blocks Spermatogenesis in Pachytene1. Biol. Reprod. 65, 1663–1671. 10.1095/biolreprod65.6.1663 11717126

[B90] Kumaresan A. Das Gupta M. Datta T. K. Morrell J. M. (2020). Sperm DNA Integrity and Male Fertility in Farm Animals: A Review. Front. Vet. Sci. 7, 1–15. 10.3389/fvets.2020.00321 32637425 PMC7317013

[B91] Lambard S. Galeraud-Denis I. Martin G. Levy R. Chocat A. Carreau S. (2004). Analysis and Significance of mRNA in Human Ejaculated Sperm from Normozoospermic Donors: Relationship to Sperm Motility and Capacitation. Mol. Hum. Reprod. 10, 535–541. 10.1093/molehr/gah064 15100385

[B92] Légaré C. Akintayo A. Blondin P. Calvo E. Sullivan R. (2017). Impact of Male Fertility Status on the Transcriptome of the Bovine Epididymis. Mol. Hum. Reprod. 23, 355–369. 10.1093/molehr/gax019 28379507

[B93] Li B. He X. Zhao Y. Bai D. Du M. Song L. (2020). Transcriptome Profiling of Developing Testes and Spermatogenesis in the Mongolian Horse. BMC Genet. 21, 1–10. 10.1186/s12863-020-00843-5 32345215 PMC7187496

[B94] Li C. Zhou X. (2012). Gene Transcripts in Spermatozoa: Markers of Male Infertility. Clin. Chim. Acta 413, 1035–1038. 10.1016/j.cca.2012.03.002 22445828

[B95] Li J. Liu X. Hu X. Tian G. G. Ma W. Pei X. (2017). MicroRNA-10b Regulates the Renewal of Spermatogonial Stem Cells through Kruppel-like Factor 4. Cell Biochem. Funct. 35, 184–191. 10.1002/cbf.3263 28436141

[B96] Li Y. Li R.-H. Ran M.-X. Zhang Y. Liang K. Ren Y.-N. (2018). High Throughput Small RNA and Transcriptome Sequencing Reveal Capacitation-Related microRNAs and mRNA in Boar Sperm. BMC Genomics 19, 736. 10.1186/s12864-018-5132-9 30305024 PMC6180635

[B97] Lian J. Zhang X. Tian H. Liang N. Wang Y. Liang C. (2009). Altered microRNA Expression in Patients with Non-obstructive Azoospermia. Reprod. Biol. Endocrinol. 7, 1. 10.1186/1477-7827-7-13 19210773 PMC2647923

[B98] Lian Y. Gòdia M. Castello A. Rodriguez-Gil J. E. Balasch S. Sanchez A. (2021). Characterization of the Impact of Density Gradient Centrifugation on the Profile of the Pig Sperm Transcriptome by RNA-Seq. Front. Vet. Sci. 8, 1–12. 10.3389/fvets.2021.668158 PMC8326511 34350225

[B99] Liu S.-S. Maguire E. M. Bai Y.-S. Huang L. Liu Y. Xu L. (2019). A Novel Regulatory Axis, CHD1L-MicroRNA 486-Matrix Metalloproteinase 2, Controls Spermatogonial Stem Cell Properties. Mol. Cel. Biol. 39. 10.1128/MCB.00357-18 PMC6362313 30455250

[B100] Liu W.-M. Pang R. T. K. Chiu P. C. N. Wong B. P. C. Lao K. Lee K.-F. (2012). Sperm-borne microRNA-34c Is Required for the First Cleavage Division in Mouse. Proc. Natl. Acad. Sci. U. S. A. 109, 490–494. 10.1073/pnas.1110368109 22203953 PMC3258645

[B101] Lu Y. Wu X. Wang J. (2019). Correlation of miR-425-5p and IL-23 with Pancreatic Cancer. Oncol. Lett. 17, 4595–4599. 10.3892/ol.2019.10099 30944648 PMC6444423

[B103] Luo J. McGinnis L. K. Carlton C. Beggs H. E. Kinsey W. H. (2014). PTK2b Function during Fertilization of the Mouse Oocyte. Biochem. Biophys. Res. Commun. 450, 1212–1217. 10.1016/j.bbrc.2014.03.083 24667605 PMC4133292

[B104] Luo L. F. Hou C. C. Yang W. X. (2016). Small Non-coding RNAs and Their Associated Proteins in Spermatogenesis. Gene 578, 141–157. 10.1016/j.gene.2015.12.020 26692146

[B105] Maciel V. L. Caldas-Bussiere M. C. Silveira V. Reis R. S. Rios A. F. L. Paes de Carvalho C. S. (2018). L-arginine Alters the Proteome of Frozen-Thawed Bovine Sperm during *In Vitro* Capacitation. Theriogenology 119, 1–9. 10.1016/j.theriogenology.2018.06.018 29958134

[B106] Martins R. P. Krawetz S. A. (2005). RNA in Human Sperm. Asian J. Androl. 7, 115–120. 10.1111/j.1745-7262.2005.00048.x 15897966

[B107] Marzano G. Chiriacò M. Primiceri E. Dell’Aquila M. Ramalho-Santos J. Zara V. (2020). Sperm Selection in Assisted Reproduction: A Review of Established Methods and Cutting-Edge Possibilities. Biotechnol. Adv. 40, 107498. 10.1016/J.BIOTECHADV.2019.107498 31836499

[B108] Michailov Y. Ickowicz D. Breitbart H. (2014). Zn2+-stimulation of Sperm Capacitation and of the Acrosome Reaction Is Mediated by EGFR Activation. Dev. Biol. 396, 246–255. 10.1016/j.ydbio.2014.10.009 25446533

[B109] Miller D. Briggs D. Snowden H. Hamlington J. Rollinson S. Lilford R. (1999). A Complex Population of RNAs Exists in Human Ejaculate Spermatozoa: Implications for Understanding Molecular Aspects of Spermiogenesis. Gene 237, 385–392. 10.1016/S0378-1119(99)00324-8 10521662

[B110] Miller D. Ostermeier G. C. Krawetz S. A. (2005). The Controversy, Potential and Roles of Spermatozoal RNA. Trends Mol. Med. 11, 156–163. 10.1016/j.molmed.2005.02.006 15823753

[B111] Miller D. Ostermeier G. C. (2006). Towards a Better Understanding of RNA Carriage by Ejaculate Spermatozoa. Hum. Reprod. Update 12, 757–767. 10.1093/humupd/dml037 16882702

[B112] Miller D. (20142014). Sperm RNA as a Mediator of Genomic Plasticity. Adv. Biol., 1–13. 10.1155/2014/179701

[B113] Miller D. (2007). Spermatozoal RNA as Reservoir, Marker and Carrier of Epigenetic Information: Implications for Cloning. Reprod. Domest. Anim. 42, 2–9. 10.1111/j.1439-0531.2007.00883.x 17688596

[B114] Mohamad S. F. S. Ibrahim S. F. Ismail N. H. Osman K. Jaafar F. H. F. Nang C. F. (2018). Quantification of HSP70 Gene Expression and Determination of Capacitation Status of Magnetically Separated Cryopreserved Bovine Spermatozoa at Different Thawing Temperature and Time. Sains Malaysiana 47, 1101–1108. 10.17576/jsm-2018-4706-04

[B115] Montjean D. De La Grange P. Gentien D. Rapinat A. Belloc S. Cohen-Bacrie P. (2012). Sperm Transcriptome Profiling in Oligozoospermia. J. Assist. Reprod. Genet. 29, 3–10. 10.1007/s10815-011-9644-3 21989496 PMC3252406

[B116] Moraveji S.-F. Esfandiari F. Sharbatoghli M. Taleahmad S. Nikeghbalian S. Shahverdi A. (2019). Optimizing Methods for Human Testicular Tissue Cryopreservation and Spermatogonial Stem Cell Isolation. J. Cel. Biochem. 120, 613–621. 10.1002/jcb.27419 30242874

[B117] Morgan H. L. Eid N. Khoshkerdar A. Watkins A. J. (2020). Defining the Male Contribution to Embryo Quality and Offspring Health in Assisted Reproduction in Farm Animals. Anim. Reprod. 17, 1–14. 10.1590/1984-3143-AR2020-0018 PMC7534566 33029211

[B118] Morrell J. Mari G. Kútvölgyi G. Meurling S. Mislei B. Iacono E. (2011). Pregnancies Following Artificial Insemination with Spermatozoa from Problem Stallion Ejaculates Processed by Single Layer Centrifugation with. Androcoll-e. Reprod. Domest. Anim. 46, 642–645. 10.1111/j.1439-0531.2010.01721.x 21114793

[B119] Neto F. T. L. Bach P. V. Najari B. B. Li P. S. Goldstein M. (2016). Spermatogenesis in Humans and its Affecting Factors. Semin. Cel Dev. Biol. 59, 10–26. 10.1016/j.semcdb.2016.04.009 27143445

[B120] Nicolas C. M. Accogli G. Douet C. Magistrini M. Vern Y. Le. Sausset A. (2020). Highlight of New Agents Inducing Capacitation-Related Changes in Stallion Spermatozoa to Cite This Version : HAL Id : Hal-02627722 Highlight of New Agents Inducing Capacitation-Related Changes in Stallion Spermatozoa. 2, 61–70.

[B121] Niu Z. Goodyear S. M. Rao S. Wu X. Tobias J. W. Avarbock M. R. (2011). MicroRNA-21 Regulates the Self-Renewal of Mouse Spermatogonial Stem Cells. Proc. Natl. Acad. Sci. 108, 12740–12745. 10.1073/pnas.1109987108 21768389 PMC3150879

[B122] Ostermeier G. C. Dix D. J. Miller D. Khatri P. Krawetz S. A. (2002). Spermatozoal RNA Profiles of normal fertile Men. Lancet 360, 772–777. 10.1016/S0140-6736(02)09899-9 12241836

[B123] Ostermeier G. C. Goodrich R. J. Moldenhauer J. S. Diamond M. P. Krawetz S. A. (2005). A Suite of Novel Human Spermatozoal RNAs. J. Androl. 26, 70–74. 10.1002/j.1939-4640.2005.tb02874.x 15611569

[B124] Ostermeier G. C. Miller D. Huntriss J. D. Diamond M. P. Krawetz S. A. (2004). Delivering Spermatozoan RNA to the Oocyte. Nature 429, 154. 10.1038/429154a 15141202

[B125] Özbek M. Hitit M. Kaya A. Jousan F. D. Memili E. (2021). Sperm Functional Genome Associated with Bull Fertility. Front. Vet. Sci. 8, 1–17. 10.3389/fvets.2021.610888 PMC8262648 34250055

[B126] Pantano L. Jodar M. Bak M. Ballescà J. L. luí. Tommerup N. Oliva R. (2015). The Small RNA Content of Human Sperm Reveals Pseudogene-Derived piRNAs Complementary to Protein-Coding Genes. RNA 21, 1085–1095. 10.1261/rna.046482.114 25904136 PMC4436662

[B127] Paradowska-Dogan A. Fernandez A. Bergmann M. Kretzer K. Mallidis C. Vieweg M. (2014). Protamine mRNA Ratio in Stallion Spermatozoa Correlates with Mare Fecundity. Andrology 2, 521–530. 10.1111/j.2047-2927.2014.00211.x 24711287

[B128] Pardede B. P. Agil M. Supriatna I. (2020). Protamine and Other Proteins in Sperm and Seminal Plasma as Molecular Markers of Bull Fertility. Vet. World 13, 556–562. 10.14202/vetworld.2020.556-562 32367964 PMC7183474

[B129] Parthipan S. Selvaraju S. Somashekar L. Arangasamy A. Sivaram M. Ravindra J. P. (2017). Spermatozoal Transcripts Expression Levels Are Predictive of Semen Quality and conception Rate in Bulls (*Bos taurus*). Theriogenology 98, 41–49. 10.1016/j.theriogenology.2017.04.042 28601154

[B130] Peifer M. (2000). Wnt Signaling in Oncogenesis and Embryogenesis-Aa Look outside the Nucleus. Sci. (80- 287, 1606–1609. 10.1126/science.287.5458.1606 10733430

[B131] Peris-Frau P. Álvarez-Rodríguez M. Martín-Maestro A. Iniesta-Cuerda M. Sánchez-Ajofrín I. Garde J. J. (2019). Comparative Evaluation of DNA Integrity Using Sperm Chromatin Structure Assay and Sperm-Ovis-Halomax during *In Vitro* Capacitation of Cryopreserved Ram Spermatozoa. Reprod. Domest. Anim. 54, 46–49. 10.1111/rda.13519 31625230

[B132] Prakash M. A. Kumaresan A. Ebenezer Samuel King J. P. Nag P. Sharma A. Sinha M. K. (2021). Comparative Transcriptomic Analysis of Spermatozoa from High- and Low-Fertile Crossbred Bulls: Implications for Fertility Prediction. Front. Cel Dev. Biol. 9, 1–14. 10.3389/fcell.2021.647717 PMC8141864 34041237

[B133] Prakash M. A. Kumaresan A. Sinha M. K. Kamaraj E. Mohanty T. K. Datta T. K. (2020). RNA-seq Analysis Reveals Functionally Relevant Coding and Non-coding RNAs in Crossbred Bull Spermatozoa. Anim. Reprod. Sci. 222, 1–25. 10.1016/j.anireprosci.2020.106621 PMC7607363 33069132

[B134] Qiu T. Wang K. Li X. Jin J. (2018). miR-671-5p Inhibits Gastric Cancer Cell Proliferation and Promotes Cell Apoptosis by Targeting URGCP. Exp. Ther. Med. 16, 4753–4758. 10.3892/etm.2018.6813 30546398 PMC6256858

[B135] Raval N. P. Shah T. M. George L. B. Joshi C. G. (2019). Insight into Bovine (*Bos indicus*) Spermatozoal Whole Transcriptome Profile. Theriogenology 129, 8–13. 10.1016/j.theriogenology.2019.01.037 30784792

[B136] Rawe V. Y. Payne C. Schatten G. (2006). Profilin and Actin-Related Proteins Regulate Microfilament Dynamics during Early Mammalian Embryogenesis. Hum. Reprod. 21, 1143–1153. 10.1093/humrep/dei480 16428331

[B138] Røsok O. Pedeutour F. Ree A. H. Aasheim H. C. (1999). Identification and Characterization of TESK2, a Novel Member of the LIMK/TESK Family of Protein Kinases, Predominantly Expressed in Testis. Genomics 61, 44–54. 10.1006/geno.1999.5922 10512679

[B139] Saacke R. G. Dalton J. C. Nadir S. Nebel R. L. Bame J. H. (2000). Relationship of Seminal Traits and Insemination Time to Fertilization Rate and Embryo Quality. Anim. Reprod. Sci. 60–61, 663–677. 10.1016/s0378-4320(00)00137-8 10844233

[B140] Sakurai K. Mikamoto K. Shirai M. Iguchi T. Ito K. Takasaki W. (2016). MicroRNA Profiles in a Monkey Testicular Injury Model Induced by Testicular Hyperthermia. J. Appl. Toxicol. 36, 1614–1621. 10.1002/jat.3326 27071960 PMC5108483

[B141] Schwab K. R. Smith G. D. Dressler G. R. (2013). Arrested Spermatogenesis and Evidence for DNA Damage in PTIP Mutant Testes. Dev. Biol. 373, 64–71. 10.1016/j.ydbio.2012.10.006 23063797 PMC3508389

[B142] Sellem E. Marthey S. Rau A. Jouneau L. Bonnet A. Perrier J.-P. (2020). A Comprehensive Overview of Bull Sperm-Borne Small Non-coding RNAs and Their Diversity across Breeds. Epigenetics Chromatin 13, 19. 10.1186/s13072-020-00340-0 32228651 PMC7106649

[B143] Selvaraju S. Parthipan S. Somashekar L. Binsila B. K. Kolte A. P. Arangasamy A. (2018). Current Status of Sperm Functional Genomics and its Diagnostic Potential of Fertility in Bovine (*Bos taurus*). Syst. Biol. Reprod. Med. 64, 484–501. 10.1080/19396368.2018.1444816 29537884

[B144] Selvaraju S. Parthipan S. Somashekar L. Kolte A. P. Krishnan Binsila B. Arangasamy A. (2017). Occurrence and Functional Significance of the Transcriptome in Bovine (*Bos taurus*) Spermatozoa. Sci. Rep. 7, 42392. 10.1038/srep42392 28276431 PMC5343582

[B145] Sendler E. Johnson G. D. Mao S. Goodrich R. J. Diamond M. P. Hauser R. (2013). Stability, Delivery and Functions of Human Sperm RNAs at Fertilization. Nucleic Acids Res. 41, 4104–4117. 10.1093/nar/gkt132 23471003 PMC3627604

[B146] Shilpa M. Selvaraju S. GirishKumar V. Parthipan S. Binsila K. B. Arangasamy A. (2017). Novel Insights into the Role of Cell-free Seminal mRNAs on Semen Quality and Cryotolerance of Spermatozoa in Bulls (*Bos taurus*). Reprod. Fertil. Dev. 29, 2446. 10.1071/RD16290 28610652

[B147] Shin J. Y. Gupta M. K. Jung Y. H. Uhm S. J. Lee H. T. (2011). Differential Genomic Imprinting and Expression of Imprinted microRNAs in Testes-Derived Male Germ-Line Stem Cells in Mouse. PLoS One 6, e22481. 10.1371/journal.pone.0022481 21799869 PMC3142150

[B148] Shirakata Y. Hiradate Y. Inoue H. Sato E. Tanemura K. (2014). Histone H4 Modification during Mouse Spermatogenesis. J. Reprod. Dev. 60, 383–387. 10.1262/jrd.2014-018 25087733 PMC4219996

[B149] Singh R. Junghare V. Hazra S. Singh U. Sengar G. S. Raja T. V. (2019). Database on Spermatozoa Transcriptogram of Catagorised Frieswal Crossbred (Holstein Friesian X Sahiwal) Bulls. Theriogenology 129, 130–145. 10.1016/j.theriogenology.2019.01.025 30844654

[B150] Sosnik J. Miranda P. V. Spiridonov N. A. Yoon S. Y. Fissore R. A. Johnson G. R. (2009). Tssk6 Is Required for Izumo Relocalization and Gamete Fusion in the Mouse. J. Cel Sci. 122, 2741–2749. 10.1242/jcs.047225 PMC2909320 19596796

[B151] Stephanie P. (2016). Characterization of Small Non-coding RNAs in the Seminal Plasma of Beef Bulls with Predicted High and Low Fertility. Electron Theses Diss. Brookings (SD): South Dakota State University.

[B152] Stiavnicka M. Alvarez O. G. Nevoral J. KraliAkova M. Sutovsky P. (2016). Key Features of Genomic Imprinting during Mammalian Spermatogenesis: Perspectives for Human Assisted Reproductive Therapy: A Review. Anat. Physiol. 6. 10.4172/2161-0940.1000236

[B153] Stowe H. M. Calcatera S. M. Dimmick M. A. Andrae J. G. Duckett S. K. Pratt S. L. (2014). The Bull Sperm microRNAome and the Effect of Fescue Toxicosis on Sperm microRNA Expression. PLoS One 9, 1–18. 10.1371/journal.pone.0113163 PMC4251976 25462855

[B154] Suh N. Blelloch R. (2011). Small RNAs in Early Mammalian Development: From Gametes to Gastrulation. Development 138, 1653–1661. 10.1242/dev.056234 21486922 PMC3074443

[B155] Suliman Y. Becker F. Wimmers K., (2018). Implication of Transcriptome Profiling of Spermatozoa for Stallion Fertility. Reprod. Fertil. Dev. 30, 1087–1098. 10.1071/RD17188 29534788

[B156] Sun Y. H. Wang A. Song C. Shankar G. Srivastava R. K. Au K. F. (2021). Single-molecule Long-Read Sequencing Reveals a Conserved Intact Long RNA Profile in Sperm. Nat. Commun. 12, 1361. 10.1038/s41467-021-21524-6 33649327 PMC7921563

[B157] Tan D. Zhou C. Han S. Hou X. Kang S. Zhang Y. (2018). MicroRNA-378 Enhances Migration and Invasion in Cervical Cancer by Directly Targeting Autophagy-Related Protein 12. Mol. Med. Rep. 17, 6319–6326. 10.3892/mmr.2018.8645 29488616 PMC5928611

[B158] Tong M.-H. Mitchell D. A. McGowan S. D. Evanoff R. Griswold M. D. (2012). Two miRNA Clusters, Mir-17-92 (Mirc1) and Mir-106b-25 (Mirc3), Are Involved in the Regulation of Spermatogonial Differentiation in Mice1. Biol. Reprod. 86. 10.1095/biolreprod.111.096313 PMC3316268 22116806

[B159] Töpfer-Petersen E. Ekhlasi-Hundrieser M. Kirchhoff C. Leeb T. Sieme H. (2005). The Role of Stallion Seminal Proteins in Fertilisation. Anim. Reprod. Sci. 89, 159–170. 10.1016/j.anireprosci.2005.06.018 16125345

[B160] Turner T. T. Bang H. J. Attipoe S. A. Johnston D. S. Tomsig J. L. (2006). Sonic Hedgehog Pathway Inhibition Alters Epididymal Function as Assessed by the Development of Sperm Motility. J. Androl. 27, 225–232. 10.2164/jandrol.05114 16278368

[B161] Turri F. Capra E. Lazzari B. Cremonesi P. Stella A. Pizzi F. (2021). A Combined Flow Cytometric Semen Analysis and miRNA Profiling as a Tool to Discriminate between High- and Low-Fertility Bulls. Front. Vet. Sci. 8, 1–12. 10.3389/fvets.2021.703101 PMC8329915 34355036

[B162] Varner D. D. Bowen J. A. Johnson L. (1993). Effect of Heparin on Capacitation/acrosome Reaction of Equine Sperm. Syst. Biol. Reprod. Med. 31, 199–207. 10.3109/01485019308988400 8274046

[B163] Varona L. Clop A. Gòdia M. Estill M. Castelló A. Balasch S. (2019). A RNA-Seq Analysis to Describe the Boar Sperm Transcriptome and its Seasonal Changes. Front. Genet. 10, 1–14. 10.3389/fgene.2019.00299 31040860 PMC6476908

[B164] Vickram A. S. Srikumar P. S. Srinivasan S. Jeyanthi P. Anbarasu K. Thanigaivel S. (2021). Seminal Exosomes – an Important Biological Marker for Various Disorders and Syndrome in Human Reproduction. Saudi J. Biol. Sci. 28, 3607–3615. 10.1016/j.sjbs.2021.03.038 34121904 PMC8176048

[B165] Vojtech L. Woo S. Hughes S. Levy C. Ballweber L. Sauteraud R. P. (2014). Exosomes in Human Semen Carry a Distinctive Repertoire of Small Non-coding RNAs with Potential Regulatory Functions. Nucleic Acids Res. 42, 7290. 10.1093/NAR/GKU347 24838567 PMC4066774

[B166] Wallrapp C. Hähnel S. Boeck W. Soder A. Mincheva A. Lichter P. (2001). Loss of the Y Chromosome Is a Frequent Chromosomal Imbalance in Pancreatic Cancer and Allows Differentiation to Chronic Pancreatitis. Int. J. Cancer 91, 340–344. 10.1002/1097-0215(200002)9999:9999<:aid-ijc1014>3.0.co;2-u 11169957

[B167] Wang H. Zhou Z. Xu M. Li J. Xiao J. Xu Z. Y. (2004). A Spermatogenesis-Related Gene Expression Profile in Human Spermatozoa and its Potential Clinical Applications. J. Mol. Med. 82, 317–324. 10.1007/s00109-004-0526-3 14985855

[B168] Wang T. Gao H. Li W. Liu C. (2019a). Essential Role of Histone Replacement and Modifications in Male Fertility. Front. Genet. 10, 1–15. 10.3389/fgene.2019.00962 31649732 PMC6792021

[B169] Wang Y. Li X. Gong X. Zhao Y. Wu J. (2019b). MicroRNA-322 Regulates Self-Renewal of Mouse Spermatogonial Stem Cells through Rassf8. Int. J. Biol. Sci. 15, 857–869. 10.7150/ijbs.30611 30906216 PMC6429012

[B170] Wang Y. Zhou Y. Ali M. A. Zhang J. Wang W. Huang Y. (2021). Comparative Analysis of piRNA Profiles Helps to Elucidate Cryoinjury between Giant Panda and Boar Sperm during Cryopreservation. Front. Vet. Sci. 8, 1–11. 10.3389/fvets.2021.635013 PMC8100531 33969033

[B171] Ward W. S. (2009). Function of Sperm Chromatin Structural Elements in Fertilization and Development. Mol. Hum. Reprod. 16, 30–36. 10.1093/molehr/gap080 19748904 PMC2790366

[B172] Watson C. N. Belli A. Di Pietro V. (2019). Small Non-coding RNAs: New Class of Biomarkers and Potential Therapeutic Targets in Neurodegenerative Disease. Front. Genet. 10, 1–14. 10.3389/fgene.2019.00364 31080456 PMC6497742

[B173] Wei X. Moncada-Pazos A. Cal S. Soria-Valles C. Gartner J. Rudloff U. (2011). Analysis of the Disintegrin-Metalloproteinases Family Reveals ADAM29 and ADAM7 Are Often Mutated in Melanoma. Hum. Mutat. 32, E2148–E2175. 10.1002/humu.21477 21618342 PMC3103704

[B174] Wu W. Hu Z. Qin Y. Dong J. Dai J. Lu C. (2012). Seminal Plasma microRNAs: Potential Biomarkers for Spermatogenesis Status. MHR Basic Sci. Reprod. Med. 18, 489–497. 10.1093/molehr/gas022 22675043

[B175] Wu Y. Xu D. Zhu X. Ren* G. Y. (2017). MiR-106a Associated with Diabetic Peripheral Neuropathy through the Regulation of 12/15-LOX-Meidiated Oxidative/Nitrative Stress. Curr. Neurovasc. Res. 14, 117–124. 10.2174/1567202614666170404115912 28393703

[B176] Wykes S. M. Visscher D. W. Krawetz S. A. (1997). Haploid Transcripts Persist in Mature Human Spermatozoa. Mol. Hum. Reprod. 3, 15–19. 10.1093/molehr/3.1.15 9239704

[B177] Xiong S. Li Y. Xiang Y. Peng N. Shen C. Cai Y. (2019). Dysregulation of lncRNA and circRNA Expression in Mouse Testes after Exposure to Triptolide. Curr. Drug Metab. 20, 665–673. 10.2174/1389200220666190729130020 31362668 PMC7062010

[B178] Xu X. Tan Y. Mao H. Liu H. Dong X. Yin Z. (2020a). Analysis of Long Noncoding RNA and mRNA Expression Profiles of Testes with High and Low Sperm Motility in Domestic Pigeons (*Columba livia*). Genes (Basel) 11, 349. 10.3390/genes11040349 PMC7230152 32218174

[B179] Xu Z. Xie Y. Zhou C. Hu Q. Gu T. Yang J. (2020b). Expression Pattern of Seminal Plasma Extracellular Vesicle Small RNAs in Boar Semen. Front. Vet. Sci. 0, 929. 10.3389/FVETS.2020.585276 PMC7685987 33263017

[B180] Yang C. C. Lin Y. S. Hsu C. C. Tsai M. H. Wu S. C. Cheng W. T. K. (2010). Seasonal Effect on Sperm Messenger RNA Profile of Domestic Swine (Sus Scrofa). Anim. Reprod. Sci. 119, 76–84. 10.1016/j.anireprosci.2009.12.002 20056359

[B181] Yang C. C. Lin Y. S. Hsu C. C. Wu S. C. Lin E. C. Cheng W. T. K. (2009). Identification and Sequencing of Remnant Messenger RNAs Found in Domestic Swine (*Sus scrofa*) Fresh Ejaculated Spermatozoa. Anim. Reprod. Sci. 113, 143–155. 10.1016/j.anireprosci.2008.08.012 18786788

[B182] Yang H.-M. Liu G. Nie Z.-Y. Nie D.-S. Deng Y. Lu G.-X. (2005). Molecular Cloning of a Novel Rat Gene Tsarg1, a Member of the DnaJ/HSP40 Protein Family. DNA Seq. 16, 166–172. 10.1080/10425170500129736 16147871

[B183] Yatsenko A. N. Georgiadis A. P. Murthy L. J. Lamb D. J. Matzuk M. M. (2013). UBE2B mRNA Alterations Are Associated with Severe Oligozoospermia in Infertile Men. Mol. Hum. Reprod. 19, 388–394. 10.1093/molehr/gat008 23378580 PMC3655768

[B184] Yatsenko A. N. O’Neil D. S. Roy A. Arias-Mendoza P. A. Chen R. Murthy L. J. (2012). Association of Mutations in the Zona Pellucida Binding Protein 1 (ZPBP1) Gene with Abnormal Sperm Head Morphology in Infertile Men. MHR Basic Sci. Reprod. Med. 18, 14–21. 10.1093/molehr/gar057 PMC3244884 21911476

[B185] Yatsenko A. N. Roy A. Chen R. Ma L. Murthy L. J. Yan W. (2006). Non-invasive Genetic Diagnosis of Male Infertility Using Spermatozoal RNA: KLHL10mutations in Oligozoospermic Patients Impair Homodimerization. Hum. Mol. Genet. 15, 3411–3419. 10.1093/hmg/ddl417 17047026

[B186] Zhai Z. Wu F. Chuang A. Y. Kwon J. H. (2013). miR-106b Fine Tunes ATG16L1 Expression and Autophagic Activity in Intestinal Epithelial HCT116 Cells. Inflamm. Bowel Dis. 19, 2295–2301. 10.1097/MIB.0b013e31829e71cf 23899543 PMC4831870

[B187] Zhang J. Zhang X. Luo H. Wang X. Cao Z. Zhang Y. (2021). Expression Analysis of Circular RNAs in Young and Sexually Mature Boar Testes. Animals 11, 1430. 10.3390/ani11051430 34067577 PMC8156704

[B188] Zhang W. Yi K. Chen C. Hou X. Zhou X. (2012). Application of Antioxidants and Centrifugation for Cryopreservation of Boar Spermatozoa. Anim. Reprod. Sci. 132, 123–128. 10.1016/j.anireprosci.2012.05.009 22673393

[B189] Zhang X. Gao F. Fu J. Zhang P. Wang Y. Zeng X. (2017). Systematic Identification and Characterization of Long Non-coding RNAs in Mouse Mature Sperm. PLoS One 12, e0173402. 10.1371/journal.pone.0173402 28291811 PMC5349675

[B190] Zhang X. Wu M. Chong Q.-Y. Zhang W. Qian P. Yan H. (2018). Amplification of Hsa-miR-191/425 Locus Promotes Breast Cancer Proliferation and Metastasis by Targeting DICER1. Carcinogenesis 39, 1506–1516. 10.1093/carcin/bgy102 30084985

[B191] Zhang Y. Dai D. Chang Y. Li Y. Zhang M. Zhou G. (2017a). Cryopreservation of Boar Sperm Induces Differential microRNAs Expression. Cryobiology 76, 24–33. 10.1016/j.cryobiol.2017.04.013 28478125

[B192] Zhao W. Ahmed S. S. Ahmed S. S. Yangliu Y. Wang H. Cai X. (2021). Analysis of Long Non-coding RNAs in Epididymis of Cattleyak Associated with Male Infertility. Theriogenology 160, 61–71. 10.1016/j.theriogenology.2020.10.033 33181482

[B193] Zhou F. Chen W. Jiang Y. He Z. (2019). Regulation of Long Non-coding RNAs and Circular RNAs in Spermatogonial Stem Cells. Reproduction 158, R15–R25. 10.1530/REP-18-0517 30939448

[B194] Ziyyat A. (2001). Differential Gene Expression in Pre-implantation Embryos from Mouse Oocytes Injected with Round Spermatids or Spermatozoa. Hum. Reprod. 16, 1449–1456. 10.1093/humrep/16.7.1449 11425828

